# Optimizing the train timetable in a high-speed rail corridor: The implications on departure time, fare cost and seat preference of passengers

**DOI:** 10.1371/journal.pone.0326170

**Published:** 2025-06-18

**Authors:** Zhipeng Huang, Limin Yang, Jinlian Li, Tao Zhang, Zixian Qu, Yusen Miao

**Affiliations:** 1 School of Traffic and Transportation, Lanzhou Jiaotong University, Lanzhou, China; 2 Key Laboratory of Railway Industry on Plateau Railway Transportation Intelligent Management and Control, Lanzhou Jiaotong University, Lanzhou, China; 3 Ministry of Science and Information Technology, China Railway Lanzhou Bureau Group Co., Ltd, Lanzhou, China; Stellenbosch University, SOUTH AFRICA

## Abstract

High-speed railway timetables are typically based on origin-destination (OD) passenger demand, establishing departure times and intervals for trains. Utilizing this data, operators systematically develop daily train timetables that are consistent across a defined operational cycle. However, this approach often overlooks individual passenger preferences for departure times, fares, and seat classes, leading to low occupancy rates for some trains while others remain difficult to book. In this article, with the number of trains predetermined and considering the diverse demands of passengers, we addresses these challenges by analyzing passenger preferences and optimizing train stopping patterns and adjacent train departure intervals. We propose a time-space-state three-dimensional network (TSSN) that integrates preferences for travel time, fares, and seat classes. Impedance functions for various network arcs are developed, incorporating these three key attributes of travel demand and transforming the passenger travel choice issue into a path selection problem within the TSSN. A bi-level programming model is formulated: the upper level optimizes train operations and fare structures, while the lower level employs user equilibrium (UE) theory to distribute OD passenger demands across trains. Using the Lanzhou-Xi’an high-speed railway corridor as a case study, we apply a genetic algorithm combined with a nested Frank-Wolfe method to solve the model. The resulting timetable balances the interests of high-speed rail operators and passengers, incorporating non-uniform departure intervals to better meet diverse travel needs. Ultimately, this approach enhances the scientific rigor and practicality of high-speed railway scheduling while accommodating passenger preferences effectively.

## 1. Introduction

The train timetable is the plan of each train’s departure, arrival, and dwell times at the origin, destination, and all intermediate stations within a cycle. It is essential for the normal operation of the high-speed rail corridor. Before the rise of high-speed railways, the train service provided by the railway system was quite limited, typically offering only one or two trains operating between the same OD pair. The variety of train types was limited, and the operating times were very fixed, which resulted in people’s personalized travel demands not being met, and their travel choices were severely limited. As high-speed railways have rapidly developed, a range of train services during multiple time periods are now gradually offered between the same OD pairs, and people’s travel choices have gradually diversified. Furthermore, the high-speed railway system has gradually become the primary choice for travel due to its fast speed, strong punctuality, eco-friendly nature, and comfortable, convenient travel experience. However, the market for passenger travel is not infinite, and competition between railways and other modes of transport has become more fierce as they vie for market share. Enhancing the competitiveness of the railway system necessitates a heightened focus on the personalized travel demands of passengers and the development of a more rational timetable. An effective train schedule not only elevates the quality of railway services and augments the operating revenue of railway operators but also significantly boosts passenger travel satisfaction. To precisely align with the burgeoning and diverse travel demands and to bolster the railway system’s competitive edge, the optimization of high-speed train schedules has become an imperative issue that demands urgent attention.

With the evolution of the railway system, the advent of high-speed railways has led to a focus on the refinement of train timetable research. The scholarly investigation into high-speed railway timetables typically encompasses several distinct categories: the real-time train scheduling problem, the introduction of new train operation lines, the collaborative optimization of train timetables, and the optimization of train timetables in response to demand variations.

Trains are inevitably subject to operational disruptions that can render pre-arranged schedules impracticable. In such instances, it is imperative to implement real-time adjustments to the affected train’s operational plan, as well as to those of the subsequent trains, in an effort to mitigate the impact of the disruption. D’Ariano et al. [1] only considered the delay of trains after interference and regarded the real-time scheduling problem of railway trains as a huge workshop scheduling problem without storage constraints. Corman et al. [2] and Altazin et al. [3] conducted the study to minimize both delay times of train and waiting times of passenger. For different targets, D’Ariano et al. [4] and Corman et al. [5] adopted an advanced decision support system ROMA (Railway traffic Optimization by Means of Alternative graphs), providing a solution for minimizing train delays. Yalçınkaya and Bayhan [6] proposed a feasible framework for generating stochastic simulation timetables that can handle perturbations occurring in railway systems promptly. Meanwhile, Kecman et al. [7] pioneered four macro-models to address the limitations of micro-models in dealing with real-time train scheduling problems. In order to improve the rate of model solving, Krasemann [8] developed a fast-solving greedy algorithm, Chen et al. [9] proposed an innovative improved algorithm (DE_JRM), Wang et al. [10] reconstructed the position vectors and the genetic evolution operator, and proposed a particle swarm optimization algorithm based on the genetic algorithm. These algorithms allow delays to be resolved quickly, greatly reducing the scope of delay propagation and meeting the requirements of real-time traffic.

The train timetable problem is already an intractable NP-hard problem [11], while the problem of adding new train operation lines links the new and existing lines, making this problem even more complicated. Burdett et al. [12] described the problem of adding new train operation lines as a hybrid job shop scheduling problem with time window constraints. Tan [13] viewed the new train line problem as a combination of the timetable problem and the rescheduling problem. In response to this problem, Flier et al. [14] considered the expected risk of train delays when adding new trains to the original timetable. Cacchiani et al. [15] studied the introduction of as many trains as possible to maximize the benefits based on a timetable as close as possible to the ideal timetable. With different research objects, there are also differences in the goals considered. Jiang et al. [16] studied dual parallel rail transit lines to minimize the travel time of additional trains and reduce the frequency of initial trains. Jiang et al. [17] investigated the problem of adding new train lines on a congested two-lane railway in the context of the Beijing-Shanghai high-speed railway corridor, taking into account the influences of station-hopping and increased dwelling time. Similarly, Gao et al. [18] also took the high-speed railway corridor as the research object and proposed a three-stage optimization method to solve and obtain a suitable train schedule.

After incorporating the elements of route planning into train timetable problems, various collaborative optimization problems have emerged. Meanwhile, as comprehensive transportation systems develop by leaps and bounds, the collaborative optimization of various transportation modes has become a hot research topic currently. Among the cooperative optimization problems, the most famous one is the cooperative optimization of train timetables and stops. Goossens et al. [19] innovatively proposed a solution model with different parking patterns based on the scheme of stops at each station. Jamili and Aghaee [20] and Jiang et al. [17] researched the jump-stop mode of trains. The former worked out a more robust stopping scheme, while the latter increased the number of trains through skip-stop to satisfy more transportation needs. Luo et al. [21] and Zhang et al. [22] focused on express and local train stopping modes to optimize the train timetable. Powered by multimodal transportation, scholars have begun to consider the synergies between different modes of transportation, especially with air-rail intermodal transportation being the most common research. Román et al. [23] and Li et al. [24] examined the development of air-rail intermodal transportation, taking into account passenger preferences. Ke et al. [25,26] and Jiang et al. [27] focused on connecting air and rail timetables to minimize the transit penalties for passengers. Among them, Jiang et al.also considered the impact of train capacity, oversaturation conditions, and station-hopping patterns.

The ultimate goal of optimizing train timetables is to maximize the convenience of passengers’ travel and meet their travel needs. Nevertheless, most of the early problems with timetable optimization were considered from the supply side, which Canca et al. [28] realized relatively early on. Therefore, they fitted the train arrival and departure times to the dynamic behavior of demand to build a detailed model that takes passenger demand into account. Li et al. [24] went a step further and conducted passenger demand forecasting when considering air-rail intermodal transportation, and Jiang et al. [27] also studied the synergistic services of air and high-speed rail in a demand-oriented manner. Shang et al. [29] and Yin et al. [30] considered the uncertainty and dynamics of passenger demand and researched ways to minimize the total passenger travel time. Most of the aforementioned studies of demand-oriented timetable models have assumed an idealized situation where the service order is fixed and there is no overtaking between trains. Considering this situation, Li et al. [31] developed four timetable models with different service sequences and overtaking conditions, and discussed the combined effects of different service orders and overtaking. Zhou et al. [32] combined passenger demand with booking decisions, both of them and Li et al. [33] have established a bilevel programming model to simultaneously optimize train routes and passenger trips. Bersani et al. [34] and Cacchiani et al. [35] also proposed a dynamic train timetable that can be modified to satisfy the demands of different passengers.

In conclusion, an abundance of research on train timetables has been conducted, spanning a wide array of topics and objectives, and considering numerous influencing factors. The principal aims of train timetable optimization research are generally focused on enhancing time efficiency and reducing costs, which serve as the most immediate and intuitive measures for evaluating the quality of outcomes. However, many of these studies have only delved into the superficial aspects, failing to adequately address the variability in passengers’ preferences for departure times, ticket pricing, and seat features, thereby overlooking more detailed analyses. As a result, operational realities often reveal a paradox where certain seat categories on specific trains may have low occupancy rates at certain times, yet tickets are hard to come by at other times. To bridge this gap and maximize the profitability of railway transportation, this paper introduces considerations of train fares and seat attributes, while also taking into account the departure times of trains. Driven by the diverse needs of passengers, a bi-level programming model is proposed that integrates both train departure times and seat classes. This holistic approach rectifies the shortcomings of previous studies and accommodates the diverse needs of passengers. Table 1 presents a systematic comparison of the focus areas, optimization objectives, models, and solution algorithms of the aforementioned timetable studies.) Based on the comparison, the main contributions of this paper are summarized as follows.

**Table 1:** Comparison of related studies with our work.

Publication	Research problem	Objective	Model	Solution approach
D’Ariano et al. (2007)	The real-time conflict resolution problem	Minimize the maximum secondary delay	AG	Branch and bound
Chen et al. (2015)	The real-time train rescheduling problem	Minimize the weighted average delay	MIP	DE-JRM
Jiang et al. (2014)	The scheduling additional train unit services problem	Minimize travel times of additional trains and shifts of initial trains	MIP	CPLEX
Tan (2015)	The adding train paths problem	Minimize the total adjustments for initial trains and the number of required train-sets	MIP	Branch and bound
Luo et al. (2018)	The train stopping schedule problem	Minimize the total passenger travel time	0-1 integer programming model	GA
Ke et al. (2020)	Railway timetable problem in AH integration service	Maximum the number of synchronizations and the coverage of synchronized flights, and minimize the penalty of passengers	Multi-objective model	CPLEX
Jiang et al. (2022)	Timetable problem considering dynamic passenger demand	Minimize the passenger waiting time along the whole line	INLP	MNA
Zhou et al. (2019)	The demand-oriented timetable problem integrated with passengers’ booking decisions	Minimize passengers’ total travel cost	BLP	A priority-based heuristic
This paper	The passengers’ differentiated demand-oriented train timetabling problem	Maximum operating income of railway companies and minimum generalized cost of passenger travel	BLP	GA with nested F-W method

- Based on the consideration of train arrival and departure times, the influence of fare and seat attributes on passengers’ travel choices is emphasized, where seat attributes are visualized through ticket prices, taking into account passengers’ travel preferences in a more detailed way.
- A bi-level programming model is proposed to determine the train stopping scheme and allocate the passenger flow demand, where the upper model optimizes the train timetable and the lower model allocates the passenger flow. The model effectively reflects the dynamic game process between train operation and passenger travel.
- A three-dimensional network of time-space-state (TSSN) is designed to reflect more intuitively the spatiotemporal information of trains, fare, and seat attributes. In this context, the state refers to the specific combination of variables that characterize the status of a train at any given moment, including the train’s location, time, and available seats. The entire process of passenger travel was cleverly transformed into a path optimization problem in the three-dimensional network, laying the foundation for introducing user equilibrium (UE) methods and the Frank-Wolfe algorithm, which has significantly optimized the train timetable.

## 2. Problem statements

### 2.1. Problem description

In today’s society, with the acceleration of urbanization and the increasingly frequent regional economic exchanges, people’s travel demands are constantly growing. High – speed rail, as an efficient and convenient transportation mode, plays an increasingly important role in the passenger transportation system. In recent years, the high – speed rail network has been continuously expanded, with more and more lines being extended and cities being incorporated into the high – speed rail transportation circle, bringing great convenience to people’s travel.

However, during peak hours, the high – speed rail transportation system faces severe congestion challenges. The congestion during peak hours is, on the one hand, due to the concentrated travel of a large number of passengers, resulting in a sharp increase in transportation demand within a short period, far exceeding the designed carrying capacity of the high – speed rail system. For example, around holidays and during the weekday commuting rush hours, the business travel population and the leisure travel population overlap, causing the passenger flow on specific lines and at stations to experience explosive growth. On the other hand, the existing high – speed rail timetables have limitations in dealing with peak – hour passenger flows. The arrangements of some train trips fail to fully consider the passenger flow change rules at different times, leading to a situation where the train capacity cannot meet the demand during peak hours, while there is idle capacity during non – peak hours. This imbalance between supply and demand further exacerbates the congestion problem, not only reducing passengers’ travel experiences but also having a negative impact on the economic and social benefits of high – speed rail operations.

Against this background, conducting research on high – speed rail timetable optimization is of great practical significance. From the perspective of passengers’ travel experiences, a reasonable optimization of the timetable can reduce passengers’ waiting time and total travel time. By accurately matching the train operation time with passengers’ travel demands, passengers can plan their trips more conveniently, reduce waiting time at stations, and improve travel efficiency. For example, for commuters, an optimized timetable can ensure that they can reach their workplaces quickly and punctually during peak hours; for tourists, it can better connect their travel arrangements and enhance travel satisfaction.

From the perspective of high – speed rail operating enterprises, optimizing the timetable helps to improve the operation efficiency of trains and the utilization rate of resources. Reasonably arranging the departure times and stopping stations of trains can reduce the dwell time of trains at stations and increase the turnover times of trains. Thus, it can increase the transportation capacity without incurring excessive operating costs, which not only improves the economic benefits of operating enterprises but also enhances their competitiveness in the transportation market.

In terms of social benefits, optimizing the high – speed rail timetable plays a positive role in alleviating traffic congestion and promoting regional economic development. An efficient high – speed rail transportation system can attract more people to choose high – speed rail for travel, reducing the pressure on road traffic, and lowering energy consumption and environmental pollution. At the same time, good high – speed rail services can strengthen the connections between regions, promoting the flow of factors such as talent, capital, and technology, and driving the coordinated development of regional economies.

**Fig 1:**
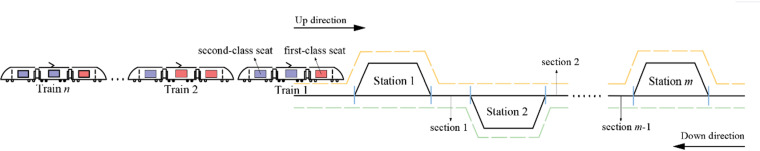
An HSR corridor with m stations and n trains.

This article studies the optimization problem of train timetables on a high-speed railway corridor with m stations running n trains, where the symbol S represents the set of stations, S={1,2,...,m}, the symbol K denotes the set of trains, K={1,2,...,n}, and the seating levels of trains are divided into first-class and second-class seats, represented by the symbol V, V={1,2}, as shown in Fig 1. There are similarities between the HSR corridor and the subway line in that there are significant imbalances in both OD passenger demand, which is manifested in the differences in passenger preference for various departure periods (in one-hour increments) within the operating timeframe, which leads to the occurrence of peak operating periods. Due to the limited space and carrying capacity of the train, there is congestion in the travel of passengers during peak hours, and the time cost of buying tickets, waiting for the train, boarding and getting off will increase, resulting in additional travel expenses. In order to achieve the purpose of travel, there will also be cases of purchasing high-grade seats at high prices. Although the ticketing income of railway transportation companies has increased at this time, it has seriously affected the travel satisfaction of passengers and is not conducive to the long-term development of railway companies.

Therefore, in order to satisfy passengers’ travel needs as well as travel preferences, we designed uneven train timetable with different departure intervals, focusing on analyzing the impact of seat class on passenger demand. To make this problem simple and easy to understand, this paper introduces the fare factor reflected by the seat class on the basis of the common time-space two-dimensional network, and designs a time-space-state three-dimensional network, which describes the whole process of the train operation with the relationship between nodes and arcs, and intuitively reflects the influence of the above factors. At the same time, in order to quantify the passenger travel cost, this paper analyzes the whole process of passenger travel, describes the generalized cost of travel, connects the passenger flow with the fare, formulates a flexible pricing strategy, establishes a bi-level programming model, explores the dynamic game process between the system optimization and the user equilibrium, and solves to obtain the equilibrium optimal train timetable.

For the established bilevel programming model, the general solution algorithm is genetic algorithm. However, considering the superiority of the Frank-Wolfe method in passenger flow distribution, this paper designs a genetic algorithm with nested Frank-Wolfe method. The solution yields train operation information such as the departure time and stopping scheme of each train, train fare information such as the unit fares of the two types of seats of all trains in each section and the fares of each OD passenger traveling on different trains and seats, and passenger allocation information such as the number of trains to which each OD passenger is assigned and the generalized cost of that OD passenger traveling on each train.

In a word, the high-speed railway train timetables optimization studied in this paper is based on the high-speed railway network structure, the number of trains, and the preference of different OD passenger flows for each departure period (in 1 hour). By comprehensively considering the impact of departure time, train unit fare, and seat class on the optimization of the train timetables, a method for optimizing decision-making is proposed.

### 2.2. Passenger travel time-space-state three-dimensional network

In the optimization of train timetable studied in this paper, the fare of the same seat class is not a fixed parameter, but a train attribute that needs to be decided according to the size of the passenger flow in the section. Moreover, the ticket prices for different seats also vary. Therefore, a three-dimensional time-space-state network (TSSN) is established in this paper, which can reflect the spatiotemporal information, fare, and seat class attributes of the train, so as to more comprehensively describe the travel selection process of passengers and the composition of their generalized travel cost.

**Fig 2:**
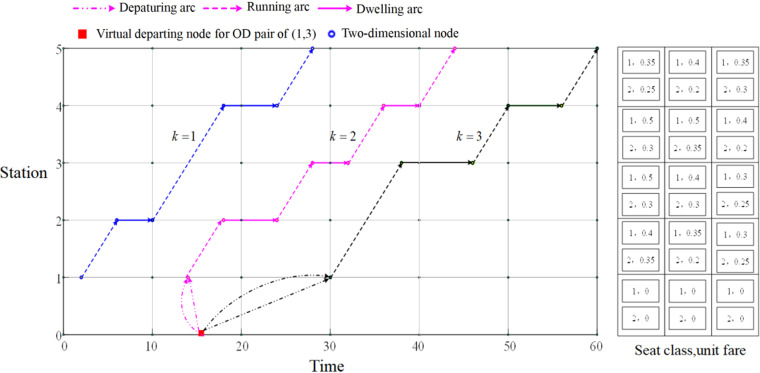
A two-dimensional extended spatiotemporal network.

In order to explain TSSN more easily, a simple example of two-dimensional extended spatiotemporal network is first used to expand the explanation, as shown in Fig 2.

On a high-speed railway corridor with 5 stations, there are 3 trains departing from station 1 in a certain hour, namely, Train k=1, k=2 and k=3. The departure time and stopping scheme of the three trains at station 1 can be obtained from the spatiotemporal network. For example, for the passengers of OD pair (1,3), they can take the train $k_{2}$ and $k_{3}$ in this paper. Based on this, this paper virtualizes an access node, and the passengers enter the space-time network from this virtual node. From this virtual departure node to the train $k_{2}$ and $k_{3}$ each has two departure arcs, which respectively represent the departure arcs of first-class seats and second-class seats. It is not difficult to find that the information of departure time and travel time can be easily obtained from the space-time network, but it is difficult to obtain the information of fare and seats. Therefore, on the right side of Fig 2, the unit fare information of two kinds of seats in different sections for 3 trains is given, but this expression is not intuitive.

**Fig 3:**
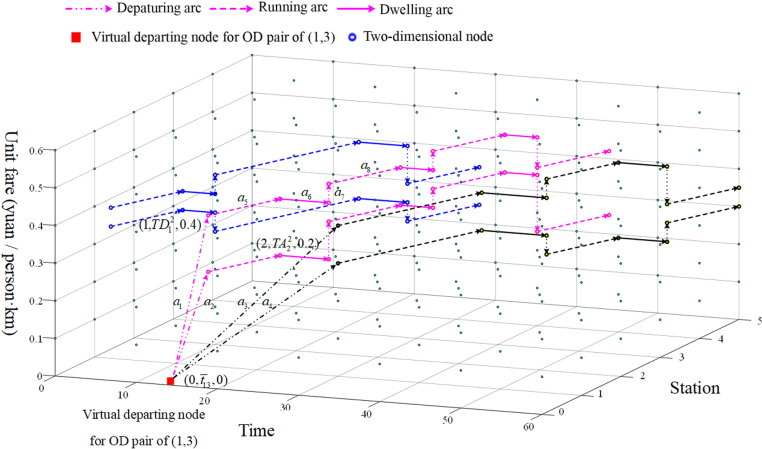
A three-dimensional TSSN of whole passenger travel process.

Therefore, this paper constructs a three-dimensional time-space-state network (TSSN) with fare as the state, as shown in Fig 3. The TSSN has three dimensions of time, space and state, which can directly reflect the passenger’s travel information (time, space, fare, and seat type). Each three-dimensional node connects the passenger’s travel state, and the three-dimensional nodes and arcs together describe the whole process of passenger travel. Table 2 lists the subscripts and parameters used in TSSN.

**Table 2:** Subscripts and parameters used in TSSN.

Symbol	Definition
G(N,A)	Three-dimensional network of passenger travel
N	Set of nodes in TSSN
A	Set of arcs in TSSN
$N^{depart}$	Set of departure nodes in TSSN, $N^{depart}\subseteq N$
$N^{arrive}$	Set of arrival nodes in TSSN, $N^{arrive}\subseteq N$
$N^{virtual}$	Set of virtual nodes in TSSN, $N^{virtual}$ ⊆N
$N^{state}$	Set of state transition nodes in TSSN, $N^{state}\subseteq N$
$A^{depart}$	Set of departure arcs in TSSN, $A^{depart}\subseteq A$
$A^{travel}$	Set of travel arcs in TSSN, $A^{travel}\subseteq A$
$A^{dwell}$	Set of dwell arcs in TSSN, $A^{stop}\subseteq A$
$A^{state}$	Set of state transition arcs in TSSN, $A^{state}\subseteq A$
i,j,r,s	Symbol of stations, i,j,r,s∈S
$t,t^{\prime }$	Symbol of time, $t,t^{\prime }\in \left[T_{S},T_{E}\right]$
$d,d^{\prime }$	Symbol of fare, $d,d^{\prime }\in \left[g_{\min}^{v},g_{\max}^{v^{\prime }}\right]$
$v,v^{\prime }$	Symbol of seat class, $v,v^{\prime }\in \left[1,2\right]$
a	Symbol of arc in TSSN, a∈A
k	Symbol of the train, k∈K
$\left(i,t,d),(j,t^{\prime },d^{\prime }\right)$	Nodes in TSSN
$\left(i,t,d;j,t^{\prime },d^{\prime }\right)$	Arc from (i,t,d) to $\left(j,t^{\prime },d^{\prime }\right)$ in TSSN, $(i,t,d;j,t^{\prime },d^{\prime })\in A$
$\omega_{i}^{k}$	If Train k stopping at Station i then $\omega_{i}^{k}=1$, otherwise $\omega_{i}^{k}=0$
${\overset{―}{t}}_{rs}$	Virtual departing time from Station r to Station s
$g_{min}^{v}$	Minimum unit fare of v -class seat, CNY/ person·km
$g_{max}^{v}$	Maximum unit fare of v -class seat, CNY/ person·km
$TA_{i}^{k}$	Arrival time of Train k at Station i
$TD_{i}^{k}$	Departing time of Train k from Station i
$TS_{i}^{k}$	Stopping time of Train k at Station i

#### 2.2.1. Three-dimensional nodes.

1. The set of arrival nodes is:

$N^{arrive}=\left\{\left(i,t,d\right)|i\in S,t=TA_{i}^{k},d=g_{i-1,t^{\prime },d^{\prime };i,t,d},\forall i,kandw_{i}^{k}=1,i\neq 1\right\}$, for example, as shown in Fig 2, $\left(2,TA_{2}^{2},0.2\right)$ indicates Train 2 arrives at Station 2 at time $TA_{2}^{2}$ with a seat class of 2. The unit fare is 0.2.

1. The set of departing nodes is:

$\begin{array}{c} N^{depart}=\left\{\left(i,t,d\right)|i\in S,t=TD_{i}^{k},d=g_{i-1,t^{\prime },d^{\prime };i,t,d},\forall i,k\;and\;w_{i}^{k}=1,i\neq m\right\} \end{array}$, and $\left(1,TD_{1}^{2},0.4\right)$ indicates Train 2 departing at Station 1 at time $TD_{1}^{2}$ with a seat class of 1. The unit fare is 0.4.

1. The set of virtual nodes is:

$N^{virtual}=\left\{\left(0,t,0\right)|t={\overset{―}{t}}_{rs},\forall r,s\in S\right\}$, and $\left(0,{\overset{―}{t}}_{13},0\right)$ represents the virtual three-dimensional node of OD pair (1,3).

1. The set of state transition nodes is:

$$\begin{array}{c} N^{state}=\left\{\left(i,t,d\right)|1<i<m,t=TD_{i}^{k},d=g_{i-1,t^{\prime },d^{\prime };i,t,d},\forall i,k\;and\;w_{i}^{k}=1\right\}. \end{array}$$

1. The set of all three-dimensional nodes is:

$N=N^{arrive}\cup N^{depart}\cup N^{virtual}\cup N^{state}$.

#### 2.2.2. Three-dimensional arcs.

1. The set of departing arcs is:

$A^{depart}=\left\{\left(0,t^{\prime },0;i,t,0\right)|i<m,t^{\prime }={\overset{―}{t}}_{is},t=TD_{i}^{k},\forall i,kand\omega_{i}^{k}=1\right\}$. Arcs of $a_{1}$ and $a_{2}$ represent departure arcs of OD pairs (1,3) of taking first-class and second-class seats by Train 2, respectively. Arcs of $a_{3}$ and $a_{4}$ represent departure arcs of OD pairs (1,3) of taking first-class and second-class seats by Train 3, respectively.

1. The set of travel arcs is:

$A^{travel}=\{\left(i,t,d;j,t^{\prime },d\right)|i,j\in S,t=TD_{i}^{k},t^{\prime }=TA_{j}^{k},d\in [g_{\min}^{v},g_{\max}^{v}],$ $\forall i,j,kand\omega_{i}^{k}\omega_{j}^{k}=1\}$, for example, the arc of $a_{5}$ is the travel arc between Station 1 and Station 2 when passengers take the first-class seat on Train 2.

1. The set of dwell arcs is:

$A^{dwell}=\left\{\left(i,t,d;i,t+TS_{i}^{k},d\right)|1<i<m,t=TA_{i}^{k},\forall i,kand\omega_{i}^{k}=1\right\}$, as shown in Fig 2, arc $a_{6}$ means that Train 2 dwells at Station 2 with the first-class seat.

1. The set of state transition arcs is:

$A^{state}=\{\left(i,t,d;i,t+TS_{i}^{k},d^{\prime }\right)|1<i<m,t=TA_{i}^{k},d=g_{i-1,i}^{k},$ $d^{\prime }=g_{i,i+1}^{k},i,kand\omega_{i}^{k}=1\}$.

1. The set of all three-dimensional arcs can be represented by $A=A^{depart}\cup A^{travel}\cup A^{dwell}\cup A^{state}$.

### 2.3. Generalized passenger travel cost

The following is an analysis of the travel cost of passengers in each link of the travel process, that is, the cost of each arc of passengers in the time-space-state three-dimensional network (TSSN). As shown in Table 3, the parameters and variables used in the generalized cost function of passenger travel are listed.

**Table 3:** Parameters and variables used to generalized cost function.

Symbol	Definition
$l_{a}$	Spatial distance of travel arc a in TSSN, $a\in A^{travel}$
$w_{a}$	Passengers’ preference degree of departing arc a in TSSN, $0\leq w_{a}\leq 1$
$\varepsilon_{D}$	Additional time for train departing
$\varepsilon_{S}$	Additional time for train stopping
α	Adjustment cost coefficient of departure time preference
$\beta_{1}^{v}$	Adjustment coefficient of flow-related cost on departure arc of v -class
$\beta_{2}^{v}$	Adjustment coefficient of flow-related cost on travel arc of v -class
$\beta_{3}^{v}$	Adjustment coefficient of flow-related cost on stopping arc of v -class
γ	Adjustment cost coefficient of dwelling time on the train
$\beta_{a}$	Adjustment coefficient of flow-related costs on arc a
$gc_{rs}^{k,v}$	Passengers’ generalized travel cost by train k and v -class seat between OD pair (r,s)
$c_{a}$	The total cost (impedance) value of arc a in TSSN, a∈A
$dc_{a}$	The cost (impedance) value of departure arc a in TSSN, $a\in A^{depart}$
$uc_{a}$	The cost (impedance) value of travel arc a in TSSN, $a\in A^{travel}$
$pc_{a}$	The cost (impedance) value of stopping arc a in TSSN, $a\in A^{dwell}$
$sc_{a}$	The cost (impedance) value of state transition arc a in TSSN, $a\in A^{state}$
$x_{a}$	Number of passengers being loaded on the arc a, $a=(i,t,d;j,t^{\prime },d^{\prime })$
$Cap_{a}$	Capacity of arc a in TSSN (number of seats)
$\delta_{rs}^{k,v,a}$	If the passenger chooses v -class seat on train k between OD pair (r,s), then $\delta_{rs}^{k,v,a}=1$, otherwise $\delta_{rs}^{k,v,a}=0$
$g_{a}$	Unit fare on arc a in TSSN

#### 2.3.1. Cost of departing arc.

**Fig 4:**
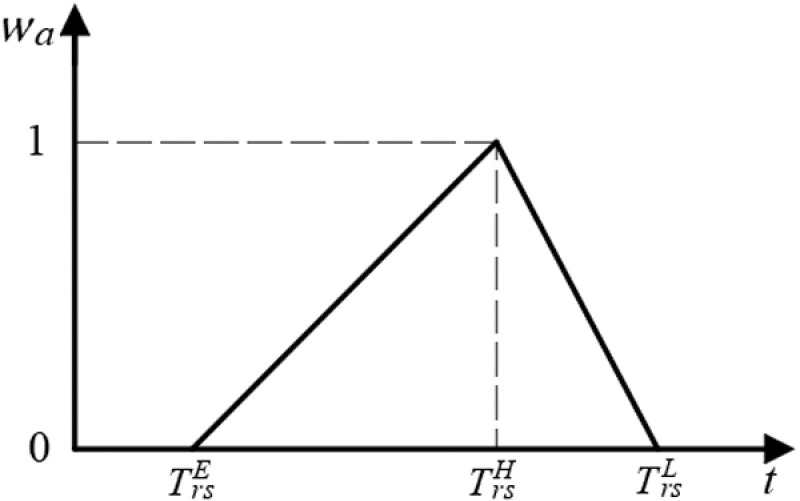
Triangular fuzzy number distribution of departure time preference.


There are significant differences in passenger preferences for departure time throughout the day. In this paper, the symbol $w_{a}$ is used to represent the preference parameter for the departure arc a in OD pair (r,s). It can be quantified by $\left(T_{rs}^{E},T_{rs}^{H},T_{rs}^{L}\right)$ obtained from data survey, where $T_{rs}^{E}$, $T_{rs}^{L}$ and $T_{rs}^{H}$, respectively, denote the earliest, latest, and most desirable departure time to passengers in OD pair (r,s). The departure time preference is represented by the triangular fuzzy number distribution as shown in Fig 4, and its membership function is shown in Eq. (1).

$$w_{a}=\left\{ \begin{array}{c} 0,t\leq T_{rs}^{E},\\ \frac{t-T_{rs}^{E}}{T_{rs}^{H}-T_{rs}^{E}},T_{rs}^{E}<t\leq T_{rs}^{H},\\ \frac{T_{rs}^{L}-t}{T_{rs}^{L}-T_{rs}^{H}},T_{rs}^{H}<t\leq T_{rs}^{L},\\ 0,t>T_{rs}^{L}. \end{array}\right.$$ (1)

Therefore, the departing arc cost (impedance) function in TSSN consist of two parts, as shown in Eq. (2), $\beta_{a}=\beta_{1}^{v},\forall a\in A,$ v∈V.


$$dc_{a}=\frac{a}{w_{a}}+\beta_{a}\cdot \frac{x_{a}}{Cap_{a}}\;\;\;\;\;\;\;\;\;\;\;\;\;\;\;\;\;\;\;\;\;\;\forall a\in A^{depart},$$ (2)

where $a/w_{a}$ denotes the passenger’s departure time preference cost, and the more passengers with a preference for departure arc a, the lower this cost is; $\beta_{a}\cdot x_{a}/Cap_{a}$ represents the flow-related cost on the departure arc a, and the more traffic loaded and the more congested arc a is, the higher the cost is.

#### 2.3.2. Cost of traveling arc.

The traveling arc cost (impedance) function in TSSN is shown in Eq. (3), $\beta_{a}=\beta_{2}^{v},\forall a\in A,v\in V$.


$$uc_{a}=g_{a}\cdot l_{a}+\beta_{a}\cdot \frac{x_{a}}{Cap_{a}}\;\;\;\;\;\;\;\;\;\;\;\;\;\;\;\;\;\;\;\;\;\;\forall a\in A^{travel},$$ (3)

where $g_{a}\cdot l_{a}$ represents the fare for the passenger’s trip, and the higher the seat class chosen by the passenger and the longer the distance traveled, the higher the total fare; Similar to the departure arc, $\beta_{a}\cdot x_{a}/Cap_{a}\;$ denotes the cost of the increase with increasing traffic on the operating arc a.

#### 2.3.4. Cost of dwelling arc.

The dwelling arc cost (impedance) function in TSSN is shown in Eq. (4), $\beta_{a}=\beta_{3}^{v},\forall a\in A,v\in V$.


$$pc_{a}=\gamma \cdot (TS_{i}^{k}+\varepsilon_{S}+\varepsilon_{D})+\beta_{a}\cdot \frac{x_{a}}{Cap_{a}}\;\;\;\;\;\;\;\;\;\;\;\;\;\;\;\;\;\;\;\;\;\;\forall a\in A^{dwell}$$ (4)

The stopping scheme of the train is crucial, but as the number of stops increases, so does the waiting time of passengers, where $\gamma \cdot (TS_{i}^{k}+\varepsilon_{S}+\varepsilon_{D})$ expresses the cost of passengers’ time on the stopping arc a including the additional hours of starting and stopping. Similarly, $\beta_{a}\cdot x_{a}/Cap_{a}\;$ reflects the cost associated with the level of congestion related to passenger flow.

#### 2.3.5. Cost of state transition arc.

As mentioned above, the state transition arc has no economic significance for passengers and is a virtual connecting arc. Hence, the impedance of this arc segment is set as $sc_{a}$ = 0.

For the convenience of representation, the impedance function of arc a in TSSN is uniformly represented by the symbol $c_{a}$ in this section. The impedance function is shown in Eq. (5).


$$\mathrm{\backslash [}c_{a}=\left\{ \begin{array}{c} dc_{a}\;\;\;a\in A^{depart}\\ uc_{a}\;\;\;a\in A^{travel}\\ pc_{a}\;\;\;a\in A^{dwell}\\ sc_{a}\;\;\;a\in A^{state} \end{array}\right.\forall a\mathrm{\backslash ]}$$ (5)

In addition, due to the capacity limitation on the arc, the cost (impedance) of the arc section a should be increased by the penalty cost due to the capacity limitation in addition to $c_{a}$. Let $u_{a}$ denote the penalty cost due to capacity limitation on arc segment a, then $u_{a}$ satisfies Eq. (6).


$$\begin{array}{c} \left\{ \;\;\;\begin{array}{c} u_{a}=0\;\;\;\;\;\;\;\;\;\;\;\;\;\;\;\;\;\;\;\;\;\;\;\;\;\;\;\;if\;\;\;\;\;\;\;\;\;\;\;\;\;\;\;\;\;\;x_{a}<\eta \cdot C\;ap_{a}\;\\ u_{a}\geq 0\;\;\;\;\;\;\;\;\;\;\;\;\;\;\;\;\;\;\;\;\;\;\;\;\;\;\;\;if\;\;\;\;\;\;\;\;\;\;\;\;\;\;\;\;\;\;x_{a}=\eta \cdot C\;ap_{a}\; \end{array}\right., \end{array}$$ (6)

where η is a number greater than 1 and $\eta \cdot C\;ap_{a}$ denotes a certain overcrowding capacity that is more realistic. If the number of passengers loaded on arc segment a is less than the capacity limit, there is no penalty charge $u_{a}$.

To sum up, the path impedance (generalized cost) of the v -class travel of the Train k selected by the passengers is shown in Eq. (7).


$$gc_{rs}^{k,v}=\sum_{a\in A}(c_{a}+u_{a})\cdot \delta_{rs}^{k,v,a}$$ (7)

## 3. Model building

This paper intends to adopt a bi-level programming method, based on the UE flow distribution principle, with the goal of maximizing the operating revenue and minimizing and equal generalized travel cost for passengers with the same travel demand, to synergistically optimize the unit fare of passengers riding different trains and seats in each section, the number of multiple units for various seat classes, and the stopping scheme and departure and arrival time of trains at each station.

### 3.1. Model structure

**Fig 5:**
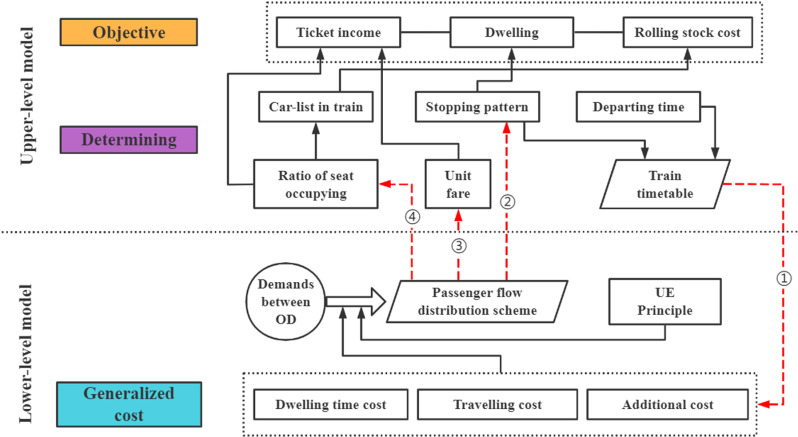
Structure of bi-level programming model.

The relationship between the upper and lower levels of the bi-level programming model established in this paper is shown in Fig 5.

The train stopping plan, and the time information of train departure and arrival decided by the upper-level model will affect the generalized cost of departure passengers.

The lower-level model distributes the OD passenger flow demand to different trains according to the generalized passenger travel cost and UE flow allocation rules. The passenger flow distribution scheme will affect the train stop pattern, unit fare, and passenger load rate. This in turn affects the optimization results of the upper-level model.

The upper-level model will adjust the unit fare, train grouping scheme, and train stopping pattern of the two kinds of seats on each train in each section according to the lower-level flow distribution results, and then adjust the train timetable. In this way, the train time information, seat information, fare information of the upper-level model and the distribution results, and generalized travel costs of the lower-level model are adjusted repeatedly until a satisfactory solution is achieved.

The train stopping plan determined by the upper – level model, as well as the departure and arrival time information of trains, will affect the generalized cost of departing passengers. The lower – level model distributes the origin - destination (OD) passenger flow demand to different trains according to the generalized passenger travel cost and user equilibrium (UE) flow allocation rules. This passenger flow distribution scheme, in turn, will affect the train stopping pattern, unit fare, and passenger load rate, thereby influencing the optimization results of the upper – level model. The upper – level model will adjust the unit fare, train grouping scheme, and the stopping patterns of two seat classes on each train in each section based on the flow distribution results of the lower – level model, and then adjust the train timetable accordingly. In this way, the train time information, seat information, fare information of the upper – level model and the passenger flow distribution results, generalized travel costs of the lower – level model are repeatedly adjusted until a satisfactory solution is obtained.

However, this framework has certain limitations. Regarding model assumptions, for the sake of simplifying the analysis, some assumptions are made in this paper. For example, certain complex factors are simplified in both the upper – and lower – level models, which may deviate from the actual situation. In reality, passengers’ travel decisions are influenced by numerous factors, such as personal emotions and unexpected events. It is difficult for the model to comprehensively consider all these factors, which may lead to inaccurate predictions of passengers’ behavior and affect the reliability of the final optimization results. In terms of data, the operation of the model highly depends on accurate input data, such as passenger flow demand, train running time, and ticket prices. However, in the actual data acquisition process, problems such as data missing, inaccuracy, or poor timeliness may occur. If data of poor quality is used, the calculation results of the model will be deviated, thereby affecting the effectiveness and practicality of the model. From the perspective of dynamic changes, the actual railway operation environment is constantly changing, including seasonal fluctuations in passenger flow and line disruptions caused by unexpected events. However, the dynamic adjustment ability of this framework is limited, and it is difficult to quickly adapt to these complex and changeable situations. It may not be able to provide effective optimization plans in a timely manner when facing unexpected situations.

Despite the above – mentioned limitations, this framework still has certain applicability in specific scenarios. For railway lines with relatively stable passenger flow demand and few external interference factors, due to their relatively simple operation environment, the impact of the assumptions and simplifications in the model on the results is small, and it can simulate and optimize the train operation plan more accurately. In some small – scale and less complex railway operation scenarios, the data required by this framework is relatively easy to obtain and process, and the computational complexity of the model is within an acceptable range, which can provide valuable decision – making references for operators. In addition, in the initial stage of railway transportation planning research, when the research focus is on establishing a basic theoretical framework and initially exploring the optimization direction, this framework can provide an effective research idea and method, helping researchers quickly build a research model and lay a foundation for further in – depth research. By analyzing the limitations and applicability of this framework, it helps to clarify the potential shortcomings of this research, provides directions for further improvement and expansion of subsequent research, and promotes the continuous development of research in the field of railway transportation optimization.

Table 4 lists the parameters and variables unique to the bi-level programming model.

**Table 4:** Unique parameters and variables in the bi-level programming model.

Symbol	Definition
$q_{rs}$	Full-day passenger flow between OD pair (r,s), unit: person
$h_{i,j}$	Pure train traveling time along the section (i,j)
$l_{i,j}$	Distance of section (i,j)
$\mu^{\max}$	Maximum train dwell time at stations
$\mu^{\min}$	Minimum train dwell time at stations
$\rho_{\min}^{section}$	Minimum interval time between adjacent trains in the section
$\rho_{\min}^{station}$	Minimum interval time between adjacent trains at the station
$E^{stop}$	Unit cost of the train stopping at the station, CNY/min
$E_{v}^{car}$	Unit operating cost for v -class seat cars, CNY/vehicle
$D_{v}$	Capacity (maximum number) of v -class seat in each car, People/vehicles
$n^{train}$	Number of car-list in each train
$\theta_{\min}^{v}$	Minimum passenger load factor of v -class seat in each train
$\theta_{\max}^{v}$	Maximum passenger load factor of v -class seat in each train
$y_{\min}^{v}$	Minimum car number with v -class seat in each train
$y_{\max}^{v}$	Maximum car number with v -class seat in each train
$\varepsilon_{a}^{k,v}$	If the seat of Train k on arc a is v -class, then $\varepsilon_{a}^{k,v}=1$, otherwise $\varepsilon_{a}^{k,v}=0$
$f_{rs}^{k,v}$	Passenger flow being distributed to v -class seats in Train k between OD pair (r,s)
$Cap_{v}^{k}$	Number of v -class seats in Train k, unit: person
$\theta_{k}^{v}$	Passenger load factor of v -class seat in Train k between origin and destination stations
$y_{k}^{v}$	Number of v -class seat cars in Train k
$TD_{1}^{k}$	Departing time of train k from original Station 1

### 3.2. Upper-level model

#### 3.2.1. Objective function.

In this paper, the maximum benefit of railway transportation is taken as the goal of upper-level planning, as shown in Eq. (8).

$\max\;\;\;\;\;\;\;\;Z_{upper}=\sum_{r,s\in S}\sum_{k\in K}\sum_{v\in V}\left(f_{rs}^{k,v}\cdot \sum_{a\in A}g_{a}\cdot l_{a}\cdot \delta_{rs}^{k,v,a}\right)-\sum_{k\in K}\sum_{v\in V}E_{v}^{car}\cdot y_{k}^{v}-$ $E^{stop}\cdot \sum_{k\in K}\sum_{i\in S}TS_{i}^{k}$ (8)

The objective function is composed of three parts. The first part represents the total ticket revenue, which is related to passenger flow, seat class, and transportation distance; The second part represents the operating cost of vehicles, which increases with the number of vehicles in the grouping and the level of seating; The third part shows the train parking cost, which is closely related to the dwell time. The railway transportation benefit is obtained by subtracting the total ticket revenue from the vehicle operating cost and the train parking cost. Of course, this does not represent the actual efficiency of rail transport, but the optimization of this has practical utility.

#### 3.2.2. Constraints.

① Fare constraint

$$g_{a}=\left\{ \begin{array}{c} g_{\min}^{v}+(g_{\max}^{v}-g_{\min}^{v})\cdot \frac{x_{a}}{Cap_{a}}\;\;\;\;\;\;\;\;\;\;\;\;\;\;\;\;\;\;\;\;\;\;\;\;\;\;\;\;\;\;if\;\;\;\;\;\;\;\;\;\;\;x_{a}\leq Cap_{a}\\ g_{\max}^{v}\;\;\;\;\;\;\;\;\;\;\;\;\;\;\;\;\;\;\;\;\;\;\;\;\;\;\;\;\;\;\;\;\;\;\;\;\;\;\;\;\;\;\;\;\;\;\;\;\;\;\;\;\;\;\;\;\;\;\;\;\;\;\;\;\;\;\;\;\;\;\;\;\;\;\;\;\;\;\;\;\;\;\;\;\;\;\;\;\;\;\;\;\;\;\;\;\;\;\;\;\;\;\;\;\;\;\;\;\;\;\;\;\;if\;\;\;\;\;\;\;\;\;\;\;x_{a}>Cap_{a}\; \end{array}\right.\;\;\;\;\;\;\;\;\;\;\;\;\;\;\;\forall a,$$ (9)

where $Cap_{a}=Cap_{v}^{k}\cdot \varepsilon_{a}^{k,v}$ represents the number of seats in the arc section a, which is the number of v -class seats of the train k related to the grouping. The unit ticket price on arc a is linearly correlated with the passenger flow $x_{a}$ loaded on it, increasing with the increase of loaded passenger flow. When the loaded passenger flow $x_{a}$ is greater than the capacity of arc a, the unit fare reaches the set maximum value $g_{max}^{v}$.

② Constraints on occupancy rates of different seat classes

$$Cap_{v}^{k}=D_{v}\cdot y_{k}^{v}\;\;\;\;\;\;\;\;\;\;\;\;\;\;\;\;\;\;\;\;\;\;\;\;\;\;\;\;\;\;\;\;\;\;\;\;\;\;\;\;\;\;\;\;\;\;\;\;\;\;\;\;\;\;\;\;\;\;\;\;\;\;\;\;\;\;\;\;\;\;\;\;\;\;\;\;\forall k$$ (10)

$$\theta_{k}^{v}=\frac{\sum_{r,s\in S}\left(f_{rs}^{k,v}\cdot \sum_{r\leq i<m}l_{i,i+1}\right)}{Cap_{v}^{k}\cdot \sum_{i\in S,i\neq m}l_{i,i+1}}\;\;\;\;\;\;\;\;\;\;\;\;\;\;\;\;\;\;\;\;\;\;\;\;\;\;\;\;\;\;\;\;\;\;\;\;\;\;\;\;\;\;\;\;\;\;\forall v,k$$ (11)

$$\theta_{\min}^{v}\leq \theta_{k}^{v}\leq \theta_{\max}^{v}\;\;\;\;\;\;\;\;\;\;\;\;\;\;\;\;\;\;\;\;\;\;\;\;\;\;\;\;\;\;\;\;\;\;\;\;\;\;\;\;\;\;\;\;\;\;\;\;\;\;\;\;\forall k$$ (12)

The number of seats of v -class on train k in constraint (10), $Cap_{v}^{k}$, is determined by the number of vehicles with v -class seats $y_{k}^{v}$ assembled in train k, and the seating capacity $D_{v}$ of vehicles with v -class seats is a known fixed value. Constraint (11) calculates the passenger load factor of v -class seats on train k between the originating and terminating stations. The calculation of passenger load factor is closely related to the distance traveled by passengers, which avoids the illusion that high volume of short-distance transportation creates a good operational efficiency, and it is better than the attendance rate, which is a common indicator of efficiency in railroads. Passenger load factor is a term used in the aviation industry to evaluate the operational efficiency of airlines. This article cites it to railway transportation, which effectively reflects the capacity utilization of railway transportation.While this index is more commonly associated with aviation, it provides a useful benchmark for understanding how effectively railway capacity is utilized. Generally speaking, when the passenger load factor reaches 60%, the transportation company can realize the profit, but the passenger occupancy rate is not the higher the better, too high will lead to the decline of service quality and the loss of passengers. Therefore, constraint (12) controls the passenger load factor within a certain range.

The passenger load factor is a more reliable indicator of capacity utilization than the attendance rate, which is a common efficiency measure in the railway industry.

While both passenger load factor and attendance rate are used to assess the efficiency of transportation systems, they measure different aspects of operational performance. The passenger load factor accounts for the capacity utilization relative to the distance traveled by passengers, while the attendance rate only reflects the proportion of seats occupied, regardless of the travel distance. Table 5 shows the difference between the occupancy rate and the attendance rate.

**Table 5:** The difference between the passenger load factor and the attendance rate.

Criteria	Passenger Load Factor	Attendance Rate
Definition	Measures capacity utilization adjusted for the distance traveled.	Measures the percentage of seats occupied on a given trip.
Scope	Reflects both the number of passengers and the distance traveled.	Only considers the number of passengers occupying seats.
Purpose	Evaluates the efficiency of capacity usage and operational performance over long distances.	Reflects the proportion of seat occupancy without considering travel distance.
Typical Application	Used in aviation and now applied to railway transportation for better capacity management.	Commonly used in railroads to measure overall occupancy rates.
Impact of Short-Distance Travel	Avoids the illusion of high efficiency in short-distance trips.	May misrepresent efficiency in cases of high short-distance travel.

Eq. (11) is used to calculate the occupancy rate of class seats on train between the originating and terminating stations. In the field of railway transportation efficiency evaluation, the traditional and commonly used attendance rate indicator has certain limitations. It cannot accurately measure the actual utilization efficiency of transportation resources. For example, a large volume of short – distance transportation may make the attendance rate data look good, but in fact, it may not fully utilize the potential of the transportation system. Therefore, this article introduces the concept of the occupancy rate, which is used in the aviation industry to evaluate the operational efficiency of airlines, into railway transportation. Theoretically, the calculation of the occupancy rate is closely related to the distance traveled by passengers, which enables it to reflect the utilization of railway transportation capacity more scientifically.

However, it must be acknowledged that although Eq. (11) has certain theoretical advantages, the research on its application to calculating the occupancy rate of railway transportation is still in the exploratory stage. In the actual railway transportation scenario, there are many complex factors. For example, the differences in passenger flow characteristics of different lines, seasonal passenger flow fluctuations, and train stopping strategies may affect the accuracy of the calculation results of Eq. (11). Currently, there is a lack of sufficient actual operation data to comprehensively verify the effectiveness of this equation in railway transportation.

To further improve the application of Eq. (11) in calculating the occupancy rate of railway transportation, future research can be carried out in the following directions: Conduct extensive field investigations to collect detailed passenger flow data from different lines and time periods, including information such as passengers’ departure locations, destinations, and ticket purchase times. Use statistical methods to conduct in – depth analysis of the collected data, and establish an occupancy rate calculation model that is more in line with the actual situation of railway transportation. Fully consider various influencing factors to improve the reliability and applicability of the calculation results of Eq. (11).

③ Train grouping number constraints

$$\sum_{v\in V}y_{k}^{v}=n^{train}\;\;\;\;\;\;\;\;\;\;\;\;\;\;\;\;\;\;\;\;\;\;\;\;\;\;\;\;\;\;\;\;\;\;\;\;\;\;\;\;\;\;\;\;\;\;\;\;\;\;\;\forall k$$ (13)

$$y_{\min}^{v}\leq y_{k}^{v}\leq y_{\max}^{v}\;\;\;\;\;\;\;\;\;\;\;\;\;\;\;\;\;\;\;\;\;\;\;\;\;\;\;\;\;\;\;\;\;\;\;\;\forall k,v$$ (14)

Eq. (13) indicates that the sum of the number of vehicles with all classes of seats grouped in train k must satisfy the number of vehicles in the train’s fixed group. Constraint (14) sets the maximum and minimum value of the number of vehicles with v -class in the grouping of train k, which is more in line with the actual situation and avoids the situation that there is no grouping of vehicles of a certain class of seating or a train is full of seats of that class.

④ Train arrival and departure times constraints

$$TA_{i}^{k}=TD_{i-1}^{k}+h_{i-1,i}+\varepsilon_{D}\cdot \omega_{i-1}^{k}+\varepsilon_{S}\cdot \omega_{i}^{k}\;\;\;\;\;\;\;\;\;\;\;\;\;\;\;\;\;\;\;\;\;\;\;\;\;\;\;\forall i>1,k$$ (15)

$$TD_{i}^{k}=TA_{i}^{k}+TS_{i}^{k}\;\;\;\;\;\;\;\;\;\;\;\;\;\;\;\;\;\;\;\;\;\;\;\;\;\;\;\forall i,k$$ (16)

The two equations above constrain the arrival and departure time of the train, introducing a 0–1 variable $\omega_{i}^{k}$ to consider whether the train stops at the station and increasing the influence of additional start-stop time.

⑤ Safety interval constraints of train operation

$$TA_{i}^{k+1}-TD_{i}^{k}\geq \rho_{\min}^{station}\;\;\;\;\;\;\;\;\;\;\;\;\;\;\;\;\;\;\;\;\;\;\;\;\;\;\;\forall i,k$$ (17)

$$TD_{i}^{k+1}-TD_{i}^{k}\geq \rho_{\min}^{section}\;\;\;\;\;\;\;\;\;\;\;\;\;\;\;\;\;\;\;\;\forall i,k$$ (18)

Due to the limitation of the number of arrival and departure tracks from the station and the arrangement of train approach routes, a certain time interval is required for adjacent trains to arrive and departure at the station. At the same time, for the sake of safety, adjacent trains also need a certain time interval for tracking operation in the section.

⑥ Dwell time constraint

$$\left\{ \begin{array}{c} \mu_{\min}\leq TS_{i}^{k}\leq \mu_{\max}\;\;\;\;\;\;\;\;\;\;\;\;\;\;\;\;\;\;\;\;\;if\;\;\;\;\;\;\;\;\;\omega_{i}^{k}=1\\ TS_{i}^{k}=0\;\;\;\;\;\;\;\;\;\;\;\;\;\;\;\;\;\;\;\;\;\;\;\;\;\;\;\;\;\;\;\;\;\;\;\;\;\;\;\;\;\;\;\;\;\;\;\;\;\;\;\;\;\;\;\;\;\;\;if\;\;\;\;\;\;\;\;\;\;\omega_{i}^{k}=0 \end{array}\right.\;\;\;\;\;\;\;\;\;\;\;\;\;\;\;\;\;\;\;\;\;\;\;\;\;\;\forall i,k$$ (19)

If the train stops at the station, the dwell time should not be too short to ensure that passengers can get on and off safely. Meanwhile, the stay time should not be too long, as it will reduce passenger satisfaction with travel and also affect the operation of subsequent related vehicles.

**Fig 6:**
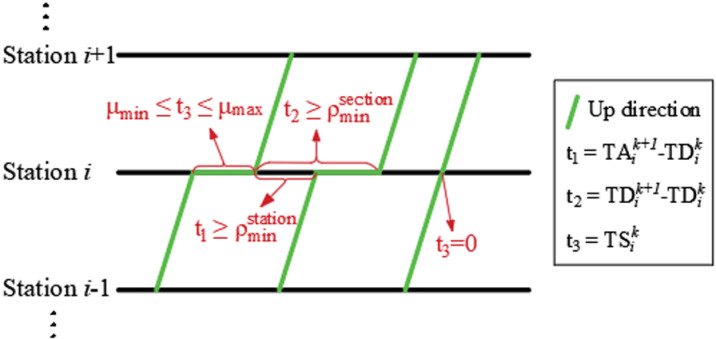
Adjacent train arrival and departure interval and dwell time constraints.

Constraints ⑤ and ⑥ are visually represented as shown in Fig 6_._

⑦ Train service capacity constraint

$$0\leq f_{rs}^{k}\leq M\cdot \omega_{r}^{k}\cdot \omega_{s}^{k},$$ (20)

where M is a sufficiently large positive number. Only when the train stops at the station, that is, $\omega_{i}^{k}=1$, passengers can get on and off the train, and the service capacity of the train can be utilized, otherwise $f_{rs}^{k}=0$.

### 3.3. Lower-level model

#### 3.3.1. Objective function.


$$\min\;\;\;Z_{lower}=\sum_{a\in A}\int_{0}^{x_{a}}c_{a}\left(\varphi \right)d\varphi$$ (21)

On the surface, it seems that the objective is to allocate traffic on the basis of minimizing the generalized cost of passenger travel to achieve the system optimum. But in fact, the objective function describes the allocation of user equilibrium optimum, which is optimal in a certain range. Its optimal value is larger than the system optimal value, but it is more in line with the actual situation and has more research and application value.

#### 3.3.2. Constraints.


$$\sum_{k\in K}\sum_{v\in V}f_{rs}^{k,v}=q_{rs}\;\;\;\;\;\;\;\;\;\;\;\;\forall r,s$$ (22)

$$f_{rs}^{k,v}\geq 0\;\;\;\;\;\;\;\;\;\;\;\;\;\;\;\;\;\;\;\;\;\;\;\;\;\;\;\;\forall r,s,k,v$$ (23)

$$x_{a}\leq \eta \cdot Cap_{a}\;\;\;\;\;\;\;\;\;\;\;\;\;\;\;\;\;\;\;\;\;\;\forall a$$ (24)

$$x_{a}=\sum_{k\in K}\sum_{r\in S}\sum_{s\in S}\sum_{v\in V}f_{rs}^{k}\cdot \delta_{rs}^{k,v,a}\;\;\;\;\;\;\;\;\;\;\;\;\;\;\;\;\;\;\;\;\;\;\forall k,v,r,s$$ (25)

Eq. (22) ensures that the passenger flow throughout the day can be allocated to the arc segment. Constraint (23) ensures that the loaded passenger traffic on the arc segment is non-negative. In constraint (24), η means the train overcrowding coefficient is a number greater than 1, which can exceed 2 in severe cases, and the accumulated number of passengers on arc a must not exceed the train capacity $\eta \cdot C\;ap_{a}$. Eq. (25) sums up the passenger flow of the passengers riding on the train to obtain $x_{a}$.

The lower-level programming model is a balanced flow distribution model with capacity constraints, which is equivalent to Wardrop’s first principle.

### 3.4. Model complexity analysis

Next, we aim to discuss the model complexity for a straightforward understanding. There are two kinds of variables in the proposed bi-level programming model. The first type refers to decision variables (i.e., ${TD}_{i}^{k}$ ${TS}_{i}^{k}$ and $f_{rs}^{k,v}$). The second type is associated with intermediate variables, i.e., ${TA}_{i}^{k}$, $\omega_{i}^{k}$, $Cap_{v}^{k}$, $\theta_{k}^{v}$, $y_{k}^{v}$, $x_{a}$, $Cap_{a}$ and $\varepsilon_{a}^{k,v}$, which also make the calculation process of the model more complicated. Additionally, in the constraints, these changing intermediate variables make the corresponding symbol ranges variable, leading constraints to be nonlinear. The model established in this paper is a bi-level programming model, and even if both the upper-level and lower-level problems are convex, the overall problem may still exhibit non-convexity. It further increases the solving difficulty, numbers of variables and constraints in the model is shown in Table 6.

**Table 6:** Numbers of variables and constraints in the model.

Variables or constraints	Total number
Integer variable ${TD}_{i}^{k}$	m·n
Integer variable ${TS}_{i}^{k}$	m·n
Integer variable $f_{rs}^{k,v}$	m·(m−1)·n
Integer variable ${TA}_{i}^{k}$	m·n
Binary variable $\omega_{i}^{k}$	m·n
Integer variable $Cap_{v}^{k}$	2·n
Continuous variable $\theta_{k}^{v}$	2·n
Integer variable $y_{k}^{v}$	2·n
Integer variable $x_{a}$	$m\cdot {\left(m-1\right)}^{2}$
Integer variable $Cap_{a}$	$m\cdot {\left(m-1\right)}^{2}$
Binary variable $\varepsilon_{a}^{k,v}$	$2\cdot m\cdot {\left(m-1\right)}^{2}\cdot n$
Constraint (9)	$m\cdot {\left(m-1\right)}^{2}$
Constraint (10)-(11)	2·n
Constraint (19)	m·n
Constraint (24)-(25)	$m\cdot {\left(m-1\right)}^{2}$

## 4. Solution algorithm

Since genetic algorithm (GA) is more compatible with the upper-level model proposed in this paper and has excellent global convergence, genetic algorithm is used to solve the upper-level model in this paper. For the lower-level model, the equilibrium allocation of passenger flow can be solved efficiently by the Frank-Wolfe method. Therefore, in this paper, the Frank-Wolfe method is embedded into the genetic algorithm for successive iterations, and the optimal train timetable and passenger flow distribution scheme can be obtained at the same time.

**Fig 7:**
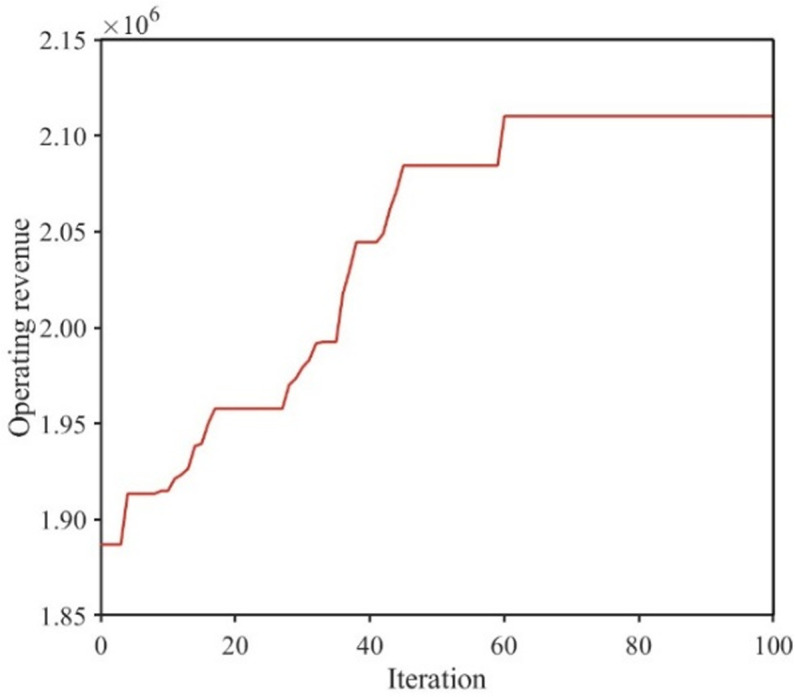
The iterative process of genetic algorithm in the upper-level model.

The decision variables in this paper involve the departure, arrival and dwell time of trains at each station. A change in one of these variables leads to a change in a series of variables. Compared to other heuristic algorithms, genetic algorithm offers a simple encoding mechanism, where interrelated variables can be encoded within the same gene segment. During the iterative process, genetic algorithm does not evaluate whether the value at a specific gene position satisfies constraints individually; instead it can evaluate a correlated group of gene values simultaneously. In this paper, genetic algorithm is used to solve the upper-level model. For the lower-level model, Frank-Wolfe method is used to solve the problem of equilibrium allocation of passenger flow. In the numerical example provided in Part 5 of this paper, the iterative process of the genetic algorithm for solving the upper-level model is illustrated in Fig 7. As shown in the figure, the genetic algorithm begins to converge at the 60th iteration. Therefore, the genetic algorithm combined with a nested Frank-Wolfe method (GAFW) proposed in this paper is effective. Genetic algorithm has been widely used in the field of train timetable optimization (e.g., Niu and Zhou [36], Cao et al. [37]). However, there is no research to show which heuristic algorithm is better for solving the train timetable optimization problem.

### 4.1. Genetic algorithm of upper-level model

#### 4.1.1. Chromosome coding rules.

**Fig 8:**
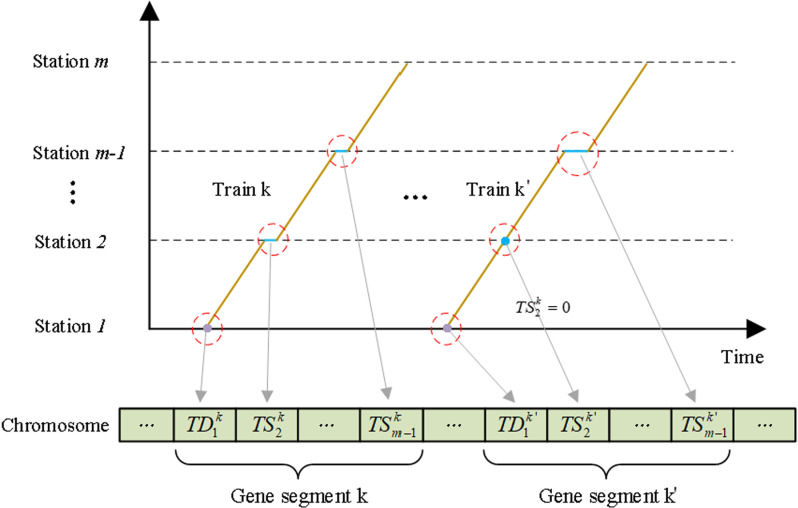
Chromosome structure and the related gene segments.

In this article, chromosomes are encoded using integers, which consists of n gene segments as shown in Fig 8. (n represents the number of trains running in the HSR corridor a day). There are ‘m-1’ genes in each segment, and they denote the departure and stopping processes of the corresponding train. Specifically, gene 1 indicates the departure time of train k from the origin station. Similarly, genes 2, 3, …, m-1 are used to represent the dwelling time at intermediate stations 2, 3, …, m-1 respectively.

To manipulate the chromosome, we construct the fitness function, which is shown in Eq. (26). $Z_{upper}$ denotes the objective value of any chromosome. $Z_{upper}^{\max}$ and $Z_{upper}^{\min}$ represent the maximum and minimum values, respectively.


$$fitness=\frac{Z_{upper}^{\max}-Z_{upper}}{Z_{upper}^{\max}-Z_{upper}^{\min}}$$ (26)

When $Z_{upper}=Z_{upper}^{\max}$, the value is 0, it means the lowest fitness degree. In contrast, fitness=1 is the highest fitness case when $Z_{upper}=Z_{upper}^{\min}$.

#### 4.1.2. Genetic manipulation.

Genetic manipulation includes selection, crossover, and mutation. By utilizing genetic manipulation, it is possible to create a new generation of populations that can lead to better evolution, resulting in a superior end solution. The specific process of gene manipulation is as follows.

1. Selecting operation

According to the fitness value, the optimal individual in the current generation remains in the next operation. In addition, the rest of the individuals will be selected randomly.

1. Crossover operation

**Fig 9:**
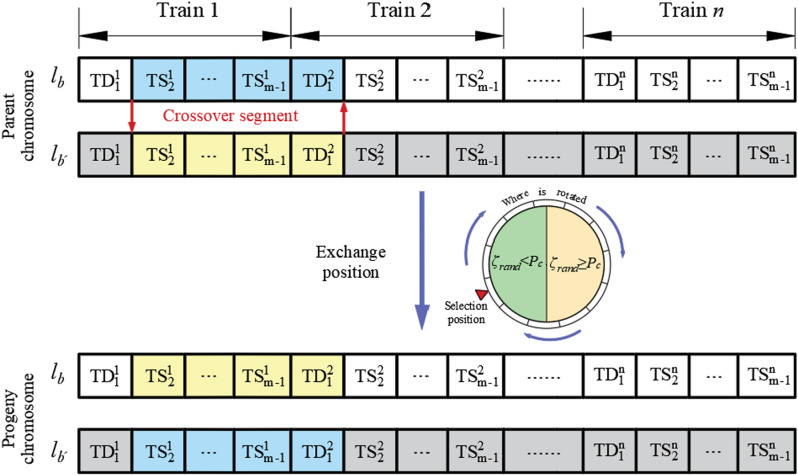
Schematic diagram of chromosome two-point crossover.

A two-point crossover operation is adopted to generate a new individual by selecting two pair chromosomes ($\ell_{b}$ and $\ell_{b^{\prime }}$) in the parent generation, then randomly determine two gene locations as intersections. By generating a random number $\zeta_{rand}\in [0,1]$, the gene information between those two points is exchanged if $\zeta_{rand}$ is less than the predetermined crossover probability $P_{c}$, otherwise remains unchanged. The detailed procedure of crossover operation is shown in Fig 9.

1. Mutation operation

Mutation operation indicates that certain genes in chromosomes mutate, and we classify them into three categories: $P_{m}^{1}$, $P_{m}^{2}$ and $P_{m}^{3}$. Where $P_{m}^{1}$ and $P_{m}^{2}$ represent the mutation probability of dwelling time and $P_{m}^{3}$ means the mutation probability of departure time. The specific mutation operation is as follows.

① If the selected gene position is $TS_{i}^{k}\in \left[\mu_{\min},\mu_{\max}\right]$ and $\zeta_{rand}<P_{m}^{1}$, then set the value of gene position $TS_{i}^{k}$ to 0.
② If the selected gene position is $TS_{i}^{k}=0$ and $\zeta_{rand}<P_{m}^{2}$, then set the value of gene position $TS_{i}^{k}$ to an integer between $\mu_{\min}$ and $\mu_{\max}$.
③ If the selected gene position is $TD_{i}^{k}$ and $\zeta_{rand}<P_{m}^{3}$, then an integer is randomly selected to replace it within the departure time range.

**Fig 10:**
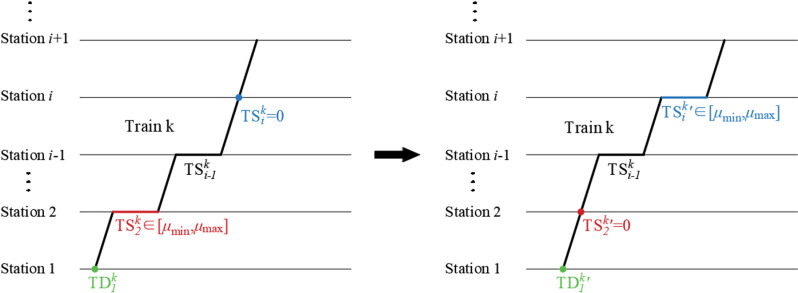
Schematic diagram of mutation operation.

The example of the above mutation operation is shown in Fig 10. When Station 2 is randomly selected as the mutation point, $TS_{2}^{k}$ becomes 0; when Station i is randomly selected as the mutation point, $TS_{i}^{k}$ becomes an integer between $\mu_{\min}$ and $\mu_{\max}$; when station 1 is randomly selected as the mutation point, $TD_{1}^{k}$ changes accordingly.

Additionally, chromosomes evolve according to the principle of meeting genetic manipulation needs. Accordingly, the gene information must obey constraint (17) and constraint (18), which can adjust chromosomes when necessary.

① Adjustment method of departure and arrival interval time at stations. For trains k and k+1, if $TA_{i}^{k+1}-TD_{i}^{k}<\rho_{\min}^{station}$, then adjust $TD_{i}^{k+1}$ with $TD_{i}^{k+1}+\nabla$, where $\nabla =\rho_{\min}^{station}-(TA_{i}^{k+1}-TD_{i}^{k})$, and increase the arrival time $TA_{i}^{k+1}$ and departure time $TD_{i}^{k+1}$ of train k+1 at all stations between station i and terminal station m by ∇.
② Adjustment method of departure interval time at stations. Similarly, if $TD_{i}^{k+1}-TD_{i}^{k}<\rho_{\min}^{\sec tion}$, then adjust $TD_{i}^{k+1}$ with $TD_{i}^{k+1}+\nabla$, where $\nabla =\rho_{\min}^{\sec tion}-(TD_{i}^{k+1}-TD_{i}^{k})$, and increase the arrival time $TA_{i}^{k+1}$ and departure time $TD_{i}^{k+1}$ of train k+1 at all stations between station i and terminal station m by ∇ at the same time.

### 4.2. Frank-Wolfe algorithm of lower-level model

Based on the timetable determined by the upper-level model, the Frank-Wolfe algorithm is updated to get the passenger flow distribution scheme ($x_{\alpha }$) in the lower-level model while ensuring that the result obeys the UE principle. Algorithm 1 shows the specific process of the Frank-Wolfe algorithm.

**Algorithm 1** Frank-Wolfe algorithm process

***% State Initialization***

1: Set the impedance $c_{a}(x_{a})=c_{a}(0)$ for all arcs in TSSN, and calculate the generalized cost $gc_{rs}^{k,v}(0)$ for each train by Eq. (7);

2: Assign the passenger flow $\left\{q_{rs}\right\}$ to trains connecting each OD pair through the all-or-nothing assignment method with the minimum value of $gc_{rs}^{k,v}(0)$ to obtain the scheme $\left\{x_{a}^{1}\right\}$;

3: Set iteration time o=1.

***% Update the cost of arcs and passenger flow distribution scheme***

4: Updating the cost of each arc $c_{a}(x_{a})$ with $x_{a}^{o}$;

5: Assign the passenger flow $\left\{q_{rs}\right\}$ to trains connecting each OD pair with the minimum value of $\delta_{rs}^{k,v,a}(x_{a}^{o})$ to obtain the new scheme {$y_{a}^{o}$};

***% Calculate the length of iterative step***

6: Calculate the iterative step ξ by $\sum_{a}\left(y_{a}^{o}-x_{a}^{o}\right)\cdot c_{a}[x_{a}^{o}+\xi \cdot (y_{a}^{o}-x_{a}^{o})]=0$.

***% Update the flow in each arc***

7: Set $x_{a}^{o+1}=x_{a}^{o}+\xi \cdot (y_{a}^{o}-x_{a}^{o}),\forall a$ to update the flow

***% End of iteration***

8: If $\sqrt{\sum_{a}{\left(x_{a}^{o+1}-x_{a}^{o}\right)}^{2}}/\sum_{a}x_{a}^{o}<\Delta$, then stop; otherwise, o=o+1 and return to Step 4.

### 4.3. Genetic algorithm flow of nested Frank-Wolfe method

**Fig 11:**
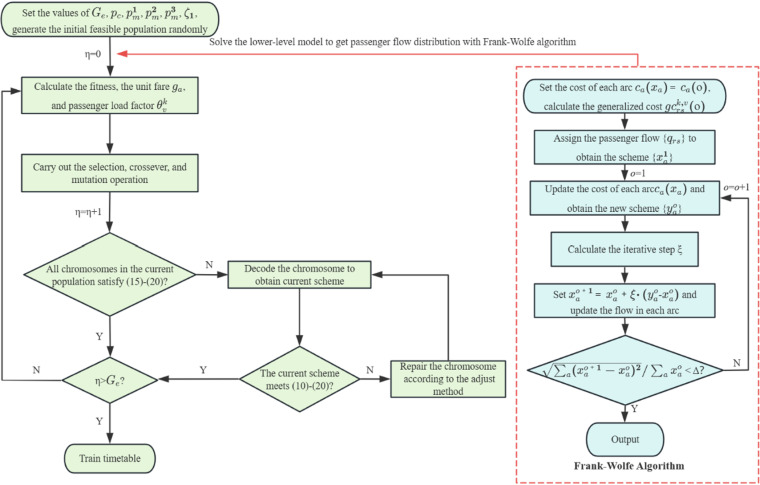
The flow of the genetic algorithm with the nested Frank-Wolfe algorithm.

In this paper, the Frank-Wolfe method is embedded in a genetic algorithm for successive iterations to simultaneously obtain the optimal train timetable and passenger flow distribution scheme. The implementation process is detailed in Algorithm 2 and Fig 11 visually illustrates the flow of the genetic algorithm with the nested Frank-Wolfe algorithm.

**Algorithm 2** Genetic algorithms with nested Frank-Wolfe method

***% Initialization***

1: Set the maximum iteration algebra Ge, the crossover probability $p_{c}$, the mutation probabilities $P_{m}^{1}$, $P_{m}^{2}$, $P_{m}^{3}$, and the cyclic variable $\varsigma_{1}=0$;

2: Generate the initial feasible solution population randomly according to the chromosome coding rules mention above;

3: Set iteration number η=0.

***% Calculate the fitness of each chromosome in the population***

4: For the current chromosome, according to the Algorithm 1, the flow distribution result $\left\{f_{rs}^{k,v}|r,s\in S,v\in V,k\in K\right\}$ of each OD pair is obtained;

5: Calculate the value of unit fare $g_{a}$ and passenger load factor $\theta_{v}^{k}$ under the current flow distribution result according to Eqs. (9) and (11);

6: Calculate the fitness of each chromosome in the current population.

***% Update population using genetic operation***

7: Carry out the selection, crossover, and mutation operation to obtain a new population and set the number of iterations η=η+1;

8: Check whether all chromosomes in the current population satisfy the Eqs. (15-20), and if so, put them into the mating pool and turn to Step 11; If not, transfer Step 9 to repair the chromosome to ensure the feasibility of the population.

***% Chromosome repair***

9: According to the current chromosome value, decode the chromosome to obtain the train timetables $\left\{TA_{i}^{k},TD_{i}^{k}|i\in S,k\in K\right\}$ and the marshaling scheme $\left\{y_{v}^{k}|v\in V,k\in K\right\}$ of vehicles with different seats;

10: Check whether the current scheme satisfies Eqs. (10-20). If it does, go to the next step, otherwise, continue to repair the chromosome according to the adjustment method.

***% Terminating testing***

11: If the iteration time η>Ge, then turn to end; otherwise, turn to Step 4.

***% End of algorithm***

### 4.4. Computational complexity analysis

Next, we aim to discuss the computational complexity for a straightforward understanding. It is obvious that the computational complexity of GAFW is mainly related to the number of stations and trains. For clarity, an example is reported here to reveal the complexity. Consider a case with m stations and n trains on a high-speed railway corridor. Each train has u departure times to choose from at the origin station. Each train has v choices of dwell time at stations from 2 to m−1. Then the computational complexity of upper-level model is $O\left(n\cdot u\cdot v^{m-2}\right)$, which can be simplified to $O\left(v^{m}\right)$. The computational complexity of lower-level model is O(λ/ε), where λ is the dimension of flow allocation and ε is the solving accuracy. Therefore, the computational complexity of bi-level programming model constructed in this paper is $O\left(v^{m}\right)$.

## 5. Numerical example

### 5.1. Parameter setting

**Fig 12:**
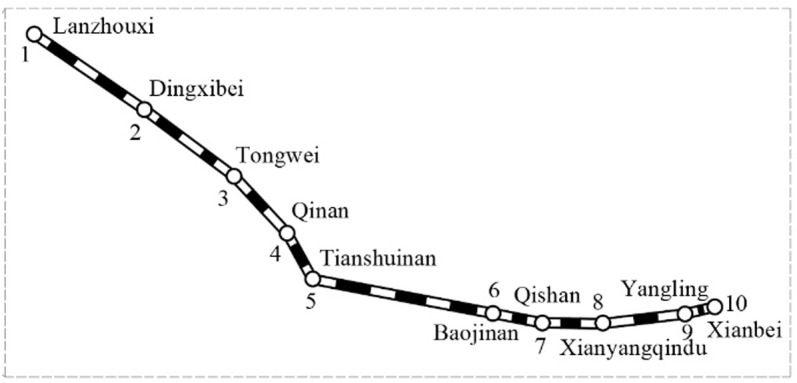
Lanzhou-Xi’an HSR corridor.

This paper takes the Lanzhou-Xi’an high-speed railway corridor (as shown in Fig 12 as the background and tests the model and algorithm proposed above. There are 10 stations and 9 sections, and each station is numbered by 1–10 from Lanzhou to Xi’an. The daily average OD passenger flow from Lanzhou West to Xi’an North as shown in Table 7.The distance and the train pure operation time of each section (excluding the additional time for train departing and stopping) are shown in Table 8. The values of other input parameters are shown in Table 9. Due to space limitations, the values of the parameter $\omega_{a}$ are presented in this paper as an example for OD pairs 1−j, as shown in Table 10. In addition, the population size $P_{size}=100$, crossover probability $P_{c}=0.8$, mutation probability $P_{m}^{1}=0.07$, $P_{m}^{2}=0.05$ and $P_{m}^{3}=0.03$, maximum iteration times Ge=100, and the convergence error limit of the lower F-W algorithm Δ=1e−3 in the genetic algorithm are considered.

**Table 7:** The daily average OD passenger flow from Lanzhou West to Xi’an North.

Station r	Station s								
	2	3	4	5	6	7	8	9	10
1	3371	1011	1686	5394	5057	1686	1349	1686	5730
2	–	450	393	674	450	168	168	225	843
3	–	–	113	168	113	56	56	113	225
4	–	–	–	225	113	56	56	168	225
5	–	–	–	–	1124	113	168	281	3933
6	–	–	–	–	–	563	843	843	2247
7	–	–	–	–	–	–	281	281	563
8	–	–	–	–	–	–	–	281	1124
9	–	–	–	–	–	–	–	–	281
Total	3371	1461	2192	6461	6855	2640	2919	3876	15170

**Table 8:** The values of distance (*l*_*i,j*_) and running time (*h*_*i,j*_) of each section *l*_*i,j*_*h*_*i,j*_.

Section (i,j)	1-2	2-3	3-4	4-5	5-6	6-7	7-8	8-9	9-10
Distance ($l_{i,j}$ /km)	103	78	55	41	141	37	41	60	35
Running time ($h_{i,j}$ /min)	26.9	20.4	14.3	10.6	36.8	9.7	10.7	15.7	9

**Table 9:** The values of other input parameters.

Parameter	Value	Parameter	Value	Parameter	Value
n	65/train	$\beta_{1}^{2}$	5	$\varepsilon_{D}$	2/min
$n^{train}$	8/Vehicle	$\beta_{2}^{1}$	0.36	$\varepsilon_{S}$	1/min
$\rho_{\min}^{section}$	3/min	$\beta_{2}^{2}$	0.4	$E^{stop}$	400/CNY per min
$\rho_{\min}^{station}$	2/min	$\beta_{3}^{1}$	0.3	$\theta_{\min}^{1}$	0.05
$y_{\max}^{1}$	2/Vehicle	$\beta_{3}^{2}$	0.5	$\theta_{\max}^{1}$	1
$y_{\min}^{2}$	6/Vehicle	α	2/CNY	$\theta_{\min}^{2}$	0.4
$y_{\min}^{1}$	1/Vehicle	γ	2/CNY per min	$\theta_{\max}^{2}$	1
$y_{\max}^{2}$	7/Vehicle	$E_{1}^{car}$	4500/CNY	$g_{\min}^{1}$	0.35/CNY per person-kilometer
$D_{1}$	60/seat	$E_{2}^{car}$	3700/CNY	$g_{\max}^{1}$	0.5/CNY per person-kilometer
$D_{2}$	80/seat	$\mu_{\max}$	6/min	$g_{\min}^{2}$	0.2/CNY per person-kilometer
$\beta_{1}^{1}$	3	$\mu_{\min}$	3/min	$g_{\max}^{2}$	0.4/CNY per person-kilometer

**Table 10:** Passengers’ preference $\begin{array}{c} w_{a} \end{array}$ for departure time of OD pairs $\begin{array}{c} \left(1,j\right) \end{array}$.

Time-period (by hours)	OD pairs								
	1-2	1-3	1-4	1-5	1-6	1-7	1-8	1-9	1-10
1	0.29	0.40	0.42	0.26	0.25	0.40	0.25	0.41	0.50
2	0.31	0.32	0.47	0.42	0.45	0.35	0.51	0.50	0.57
3	0.66	0.73	0.46	0.62	0.51	0.72	0.65	0.56	0.71
4	0.64	0.65	0.49	0.75	0.61	0.61	0.61	0.55	0.72
5	0.78	0.70	0.82	0.74	0.60	0.66	0.72	0.63	0.62
6	0.60	0.61	0.66	0.63	0.73	0.65	0.64	0.71	0.45
7	0.64	0.53	0.62	0.43	0.42	0.54	0.41	0.62	0.56
8	0.56	0.38	0.62	0.47	0.52	0.55	0.38	0.66	0.54
9	0.59	0.31	0.55	0.57	0.36	0.36	0.44	0.42	0.43
10	0.54	0.45	0.49	0.64	0.48	0.43	0.62	0.36	0.36
11	0.42	0.43	0.45	0.68	0.62	0.44	0.61	0.57	0.53
12	0.39	0.58	0.46	0.73	0.53	0.61	0.37	0.47	0.56
13	0.59	0.69	0.54	0.58	0.68	0.67	0.67	0.75	0.53
14	0.67	0.50	0.43	0.38	0.65	0.50	0.69	0.56	0.64
15	0.40	0.59	0.49	0.32	0.54	0.64	0.42	0.47	0.55
16	0.35	0.46	0.48	0.29	0.43	0.43	0.55	0.46	0.28
17	0.36	0.38	0.34	0.27	0.35	0.27	0.29	0.31	0.51
18	0.11	0.17	0.18	0.21	0.16	0.13	0.18	0.21	0.15

### 5.2. Computed results

#### 5.2.1. Upper-level model calculation results.

**Fig 13:**
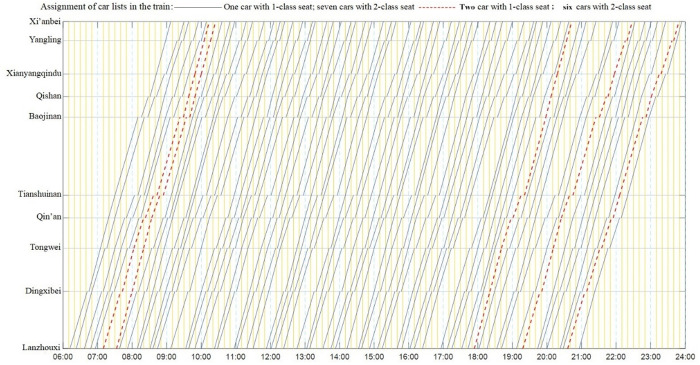
Optimized unbalanced train operation chart.

Through calculation, an optimized unbalanced train operation diagram is obtained, as shown in Fig 13. Among them, the 6th, 7th, 52nd, 58th, and 64th trains are formed into two first-class EMUs, and other trains are formed into one first-class EMU. The departure and arrival times of the train at each station, the number of stops, and the number of passengers served are shown in Table 11.

**Table 11:** Departure and arrival time of train timetable and number of passengers served.

Train NO.	Departing time	Arriving time	Traveling time	Stopping pattern of intermediate station								dwelling time	Number of passengers served	
				2	3	4	5	6	7	8	9		First-class seat	Second-class seat
1	6:12	9:07	2:55	1	0	0	0	1	1	0	0	0:12	31	481
2	6:23	9:30	3:07	0	1	0	0	1	1	1	1	0:18	59	656
3	6:37	9:38	3:01	1	0	1	1	0	0	0	1	0:15	31	886
4	6:48	9:55	3:07	1	0	1	1	0	1	0	1	0:18	34	854
5	6:56	10:09	3:13	1	1	1	0	1	1	0	1	0:21	71	904
6	7:10	10:14	3:04	1	0	1	1	1	0	0	0	0:18	109	926
7	7:33	10:25	2:52	0	0	0	1	1	0	0	0	0:12	119	629
8	7:38	10:36	2:58	1	1	1	0	0	1	0	0	0:12	42	580
9	7:52	10:50	2:58	1	0	1	0	0	1	1	0	0:12	19	535
10	8:09	10:58	2:49	0	0	0	0	1	1	0	0	0:09	15	356
11	8:14	11:18	3:04	1	1	1	0	0	0	1	1	0:15	59	490
12	8:31	11:29	2:58	0	1	0	0	0	1	1	1	0:12	12	392
13	8:36	11:49	3:13	1	0	1	1	0	1	1	1	0:21	19	739
14	8:44	12:06	3:22	1	1	1	1	1	1	0	1	0:27	47	948
15	9:07	12:17	3:10	1	0	0	1	1	1	0	1	0:21	23	503
16	9:33	12:25	2:52	0	1	1	0	0	0	0	1	0:09	15	473
17	9:38	12:39	3:01	1	1	1	0	1	0	0	0	0:15	31	500
18	9:49	12:56	3:07	1	1	1	0	1	0	0	1	0:18	6	488
19	10:00	13:13	3:13	1	1	0	1	0	1	1	1	0:21	18	732
20	10:23	13:18	2:55	0	0	1	0	1	1	0	0	0:12	6	401
21	10:28	13:38	3:10	1	0	1	1	1	0	1	0	0:21	26	760
22	10:57	13:49	2:52	0	0	1	0	0	1	1	0	0:09	27	370
23	11:02	14:03	3:01	1	0	1	0	1	1	0	0	0:15	9	379
24	11:19	14:20	3:01	0	1	0	1	0	0	1	1	0:15	16	804
25	11:30	14:28	2:58	0	0	1	1	1	0	0	0	0:15	38	689
26	11:53	14:45	2:52	1	0	0	0	0	1	1	0	0:09	28	334
27	12:01	14:56	2:55	1	0	0	1	0	0	1	0	0:12	14	452
28	12:09	15:16	3:07	1	1	0	0	1	1	1	0	0:18	8	548
29	12:23	15:30	3:07	0	1	1	1	0	1	0	1	0:18	6	657
30	12:46	15:38	2:52	1	1	0	0	0	0	0	1	0:09	7	369
31	13:03	16:04	3:01	0	0	1	1	0	1	1	0	0:15	16	622
32	13:08	16:21	3:13	1	1	1	0	1	1	0	1	0:21	28	492
33	13:31	16:26	2:55	1	0	1	0	1	0	0	0	0:12	11	533
34	13:45	16:43	2:58	1	1	0	0	0	1	1	0	0:12	12	319
35	13:53	16:54	3:01	1	1	1	0	1	0	0	0	0:15	9	544
36	14:13	17:14	3:01	0	0	1	1	0	0	1	1	0:15	20	498
37	14:33	17:28	2:55	0	0	0	0	1	1	1	0	0:12	18	504
38	14:38	17:39	3:01	1	0	1	1	0	0	0	1	0:15	10	663
39	14:52	17:50	2:58	1	1	0	0	0	1	1	0	0:12	20	342
40	15:06	18:01	2:55	1	0	0	1	0	0	0	1	0:12	11	485
41	15:14	18:21	3:07	1	1	1	1	0	0	1	0	0:18	28	602
42	15:40	18:32	2:52	0	1	1	0	0	0	0	1	0:09	13	401
43	15:48	18:43	2:55	0	1	1	0	1	0	0	0	0:12	23	682
44	16:02	19:00	2:58	1	1	0	0	0	1	0	1	0:12	9	368
45	16:10	19:20	3:10	1	1	1	0	0	1	1	1	0:18	25	444
46	16:24	19:37	3:13	0	1	1	0	1	1	1	1	0:21	38	618
47	16:56	19:42	2:46	0	1	1	0	0	0	0	0	0:06	11	342
48	17:07	19:53	2:46	1	0	0	0	0	1	0	0	0:06	19	336
49	17:15	20:07	2:52	1	1	0	0	0	1	0	0	0:09	18	366
50	17:32	20:24	2:52	1	0	0	0	0	0	1	1	0:09	6	364
51	17:43	20:38	2:55	0	0	1	0	1	0	1	0	0:12	44	658
52	17:54	20:43	2:49	0	0	1	1	0	0	0	0	0:09	132	683
53	18:02	20:57	2:55	0	1	1	0	1	0	0	0	0:12	28	696
54	18:16	21:08	2:52	0	1	1	0	0	1	0	0	0:09	19	395
55	18:33	21:25	2:52	1	0	0	0	0	0	1	1	0:09	9	310
56	18:41	21:54	3:13	1	1	1	1	0	1	1	0	0:21	122	922
57	19:07	22:05	2:58	1	0	1	0	0	1	0	1	0:12	41	492
58	19:18	22:28	3:10	1	0	0	1	1	1	0	1	0:21	128	1090
59	19:44	22:33	2:49	0	0	0	1	0	1	0	0	0:09	45	620
60	19:49	22:50	3:01	1	1	0	0	1	0	0	1	0:15	48	710
61	20:00	23:01	3:01	1	1	0	0	1	0	0	1	0:15	41	739
62	20:23	23:12	2:49	0	0	0	0	1	0	0	1	0:09	24	598
63	20:28	23:26	2:58	1	1	1	0	0	0	1	0	0:12	29	565
64	20:36	23:49	3:13	1	1	1	0	1	0	1	1	0:21	124	856
65	20:56	23:54	2:58	0	1	1	0	0	1	1	0	0:12	52	481

Note: in the stop scheme column, “1” means stop, and “0” means no stop.

In this scheme, the first train departs from the origin station at 6:12 and arrives at the terminal station at 9:07. The last train departs from the origin station at 20:56 and arrives at the terminal station at 23:54. It basically covers the operating time range of the whole day [6:00, 24:00], and can fully satisfy the travel needs of passengers in various periods.

The trains with the shortest travel time are the 47th and 48th trains, which take 2 hours and 46 minutes from the origin station to the terminal station. There are 2 stops at the intermediate station, with a parking time of 6 minutes and an additional time for train departing and stopping of 6 minutes; The train with the longest travel time is the 14th train, which lasts 3 hours and 22 minutes from origin to destination. It stops at the intermediate station for 7 stations, with a parking time of 27 minutes and an additional time for train departing and stopping of 21 minutes.

The train with the highest number of serviced passengers in the first-class seat is the 52nd train, with 132 passengers served, and the train with the lowest number of serviced passengers is the 18th train, with 6 serviced passengers; The train with the largest number of passengers served in the second-class is Train 58, and the number of serviced passengers is 1090. The train with the least serviced passengers in the second-class is Train 55, and the number of serviced passengers is 310.

**Fig 14:**
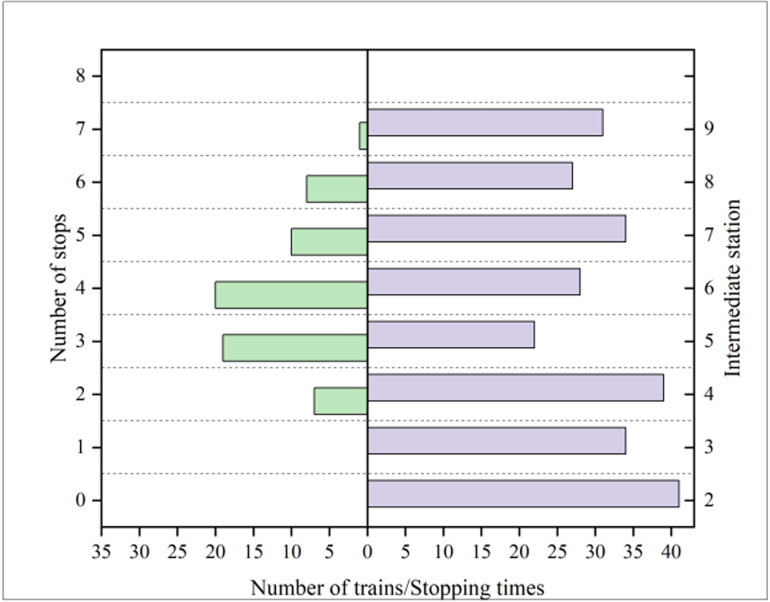
Stopping times of each train and the number of trains stopping at each intermediate station.

Statistics of train stops are shown in the left half of Fig 13. The number of trains stopping at the intermediate station for 4 times is the most, and is up to 20; There are 19 trains stopping 3 times, while there are more trains stopping 2, 5, and 6 times, with 7, 10, and 8 trains respectively; Only one train stops for 7 times; No train stops once, all stops (8 times) or all without stops (0 times). The number of stops of all 65 trains at intermediate Stations 2–9 is relatively balanced. Among them, there are more trains stopping at intermediate Stations 2 and 4, amounting to 41 and 39 trains, respectively; The number of trains stopping at the intermediate Station 5 is relatively small, with 22 trains; The stopping times of other intermediate stations are relatively balanced, as shown in the right half of Fig 14

#### 5.2.3. Lower-level model calculation results.

**Fig 15:**
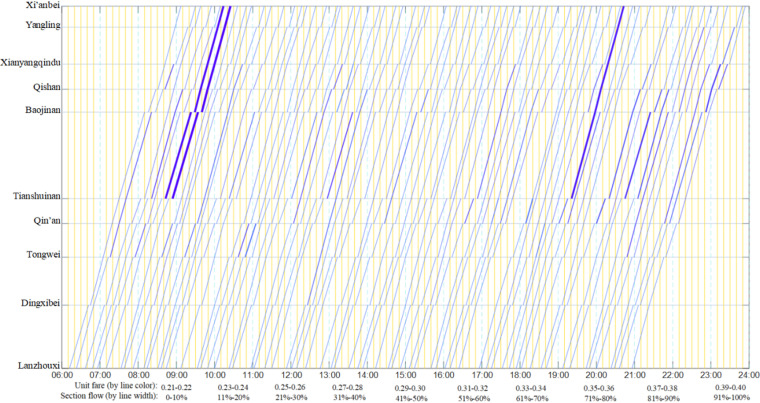
Color scale train operation diagram with first-class seat fare attribute and passenger flow distribution.

**Fig 16:**
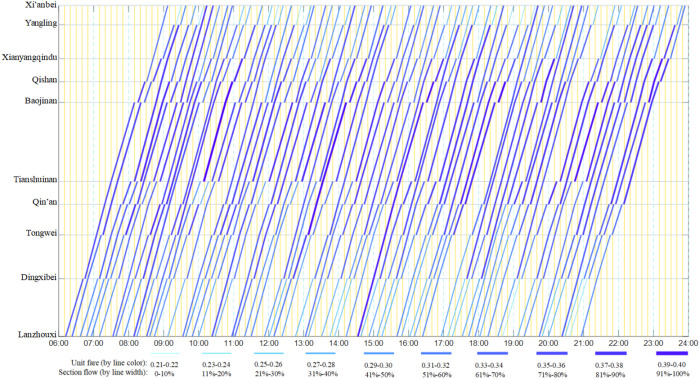
Color scale train operation diagram with second-class seat fare attribute and passenger flow distribution.

By calculation, the unit fare and flow distribution results are obtained when the passenger flow demands of all 45 OD pairs are assigned to 65 trains. Due to the large amount of data, it is represented through Figs 15 and 16. Among them, Fig 15. shows the unit fare and flow allocation of the first-class seats of each train in each operation section. It is not difficult to find that the occupancy rate of each train is low; Fig 15 shows the unit fare and flow distribution of the second-class seats of each train in each operating interval, and the flow allocation is relatively uniform.

The passenger capacity index of each train between the origin station and the terminal station is shown in Table 12, which meets the parameter setting of the numerical example. Among them, the maximum seating rate of first-class seats is 0.50, the minimum seating rate of first-class seats is 0.06, and the average seating rate of first-class seats is 0.15; The maximum seating rate of second-class seats is 0.72, the minimum seating rate of second-class seats is 0.46, and the average seating rate of second-class seats is 0.56.

**Table 12:** Occupying rate of v-class seat in each train.

Train No.	1-class	2-class	Train No.	1-class	2-class	Train No.	1-class	2-class	Train No.	1-class	2-class	Train No.	1-class	2-class
1	0.13	0.56	14	0.08	0.49	27	0.07	0.56	40	0.13	0.57	53	0.09	0.57
2	0.19	0.58	15	0.08	0.48	28	0.08	0.72	41	0.19	0.56	54	0.10	0.57
3	0.12	0.64	16	0.16	0.53	29	0.08	0.49	42	0.11	0.60	55	0.11	0.51
4	0.14	0.60	17	0.09	0.57	30	0.08	0.56	43	0.08	0.54	56	0.28	0.49
5	0.23	0.58	18	0.13	0.51	31	0.06	0.54	44	0.09	0.55	57	0.18	0.60
6	0.46	0.65	19	0.09	0.54	32	0.18	0.50	45	0.15	0.52	58	0.12	0.62
7	0.50	0.65	20	0.12	0.59	33	0.16	0.52	46	0.13	0.60	59	0.15	0.53
8	0.12	0.52	21	0.09	0.50	34	0.10	0.48	47	0.11	0.57	60	0.24	0.62
9	0.16	0.62	22	0.18	0.56	35	0.11	0.48	48	0.16	0.51	61	0.21	0.62
10	0.15	0.61	23	0.14	0.51	36	0.14	0.46	49	0.12	0.49	62	0.12	0.62
11	0.24	0.51	24	0.08	0.54	37	0.06	0.65	50	0.09	0.58	63	0.19	0.59
12	0.12	0.54	25	0.06	0.56	38	0.09	0.59	51	0.15	0.55	64	0.18	0.64
13	0.10	0.63	26	0.23	0.49	39	0.16	0.49	52	0.44	0.63	65	0.17	0.61

Table 13 shows the passenger travel information and the average generalized cost of each OD. The average time range (earliest departure time – latest departure time) for each OD passenger to take the train is 13 hours and 44 minutes. Among them, the OD pair (3,5) is the smallest in the travel time domain, which is 9 hours and 57 minutes; The largest are OD pairs (1,7) and (1,10), both at 14 hours and 44 minutes. The train running time range serving each OD calculated by this example is relatively large, which makes the travel choice range of each OD passenger larger.

**Table 13:** Passenger travel information and generalized cost mean for each OD pair.

OD pairs	Earliest departure time	Latest departure time	Number of trains	Generalized cost	OD pairs	Earliest departure time	Latest departure time	Number of trains	Generalized cost
1-2	6:12	20:36	41	41	3-10	7:16	21:49	34	178
1-3	6:23	20:56	34	71	4-5	7:50	20:0	14	23
1-4	6:37	20:56	39	95	4-6	8:15	21:55	17	77
1-5	6:37	19:44	22	114	4-7	8:1	22:9	18	96
1-6	6:12	20:36	28	168	4-8	9:5	22:9	15	117
1-7	6:12	20:56	34	188	4-9	7:50	21:55	17	141
1-8	6:23	20:56	27	208	4-10	7:50	22:9	39	155
1-9	6:23	20:36	31	231	5-6	8:43	20:45	7	65
1-10	6:12	20:56	65	246	5-7	8:21	21:5	10	79
2-3	7:29	21:9	22	33	5-8	10:9	20:20	9	99
2-4	7:10	21:9	23	57	5-9	8:10	20:45	12	121
2-5	7:10	19:51	14	74	5-10	8:10	21:5	22	139
2-6	6:45	21:9	17	130	6-7	8:16	21:31	13	24
2-7	6:45	19:51	22	149	6-8	8:27	22:52	7	43
2-8	8:25	21:9	17	168	6-9	8:27	22:52	12	67
2-9	7:10	21:9	22	193	6-10	8:16	22:52	28	83
2-10	6:45	21:9	41	207	7-8	8:42	23:12	16	23
3-4	7:55	21:49	22	29	7-9	8:42	21:46	15	48
3-5	9:43	19:40	6	48	7-10	8:31	23:12	34	61
3-6	7:16	21:35	14	102	8-9	8:59	23:18	12	28
3-7	7:16	21:49	18	120	8-10	8:59	23:29	27	43
3-8	7:16	21:49	15	141	9-10	9:21	23:40	31	18
3-9	7:16	21:35	19	164					

The average value of the generalized cost of passengers is the average value of the generalized cost of each train that passengers choose to ride, and its error meets the error range Δ=1e−3 of the flow distribution results $x_{a}$ of the two adjacent arcs set by the F-W algorithm of the lower level model. When $x_{a}$ reaches the error range, the impedance value (generalized cost) of each arc segment can be calculated accordingly. Due to space limitations, this paper does not list the generalized costs of all OD pairs of passengers traveling on each train, but only takes OD pair (3,4) as an example for illustration, as shown in Table 14. The number of trains that this OD pair passengers can take is 22, and the train serial numbers are shown in the table. Some of trains have higher generalized costs and are not loaded with traffic. The generalized cost of other trains with flow loading is: The first-class seat has a maximum of 30 and a minimum of 26; The second-class seat has a maximum of 28 and a minimum of 26. All meet the stop conditions set in the balanced flow distribution algorithm and are within the error range.

**Table 14:** Number of passengers and generalized cost for OD pair (3,4).

Train No.	1-class		2-class		Train No.	1-class		2-class	
	Number of passengers	Generalized cost	Number of passengers	Generalized cost		Number of passengers	Generalized cost	Number of passengers	Generalized cost
5	26	28	215	26	42	0	40	0	39
8	27	26	126	27	43	0	40	0	39
11	30	27	78	26	45	0	39	0	40
14	22	27	209	27	46	0	39	0	41
16	0	37	60	26	47	0	38	0	40
17	31	30	53	27	53	0	39	0	39
18	0	34	76	27	54	0	42	0	41
29	0	38	20	26	56	0	42	0	42
32	0	35	41	28	63	0	39	0	39
35	0	39	0	39	64	0	39	0	39
41	0	40	0	39	65	0	39	0	37

**Fig 17:**
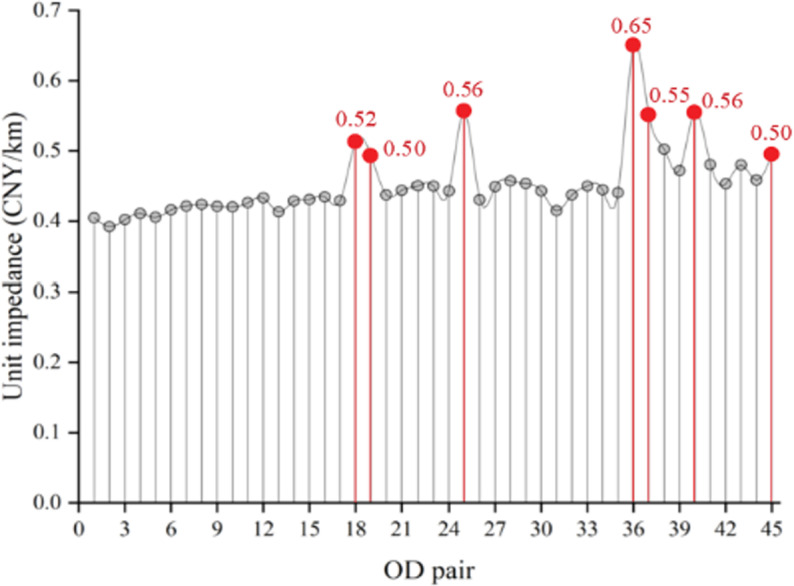
Unit impedance of each OD pair.

According to the lower-level model in this paper, the travel impedance of passengers with the same OD pair is equal (within the error range). Due to the difference of their departure preferences and the different service frequencies of trains at different stations, the generalized travel costs (impedance) of passengers with different OD pairs varies, as shown in Fig 17. Through the analysis of the calculation results, it can be found that the unit impedance difference of most OD pairs is not significant, and only individual OD pairs have higher unit impedance. As shown in the figure, the impedance of the 18th, 19th, 25th, 36th, 37th, 40th and 45th OD pairs is higher.

According to the lower-level model in this paper, the travel impedance of passengers with the same OD (Origin-Destination) pair is equal (within the error range). However, due to differences in passengers’ departure preferences and the varying service frequencies of trains at different stations, the generalized travel costs (impedance) of passengers with different OD pairs vary, as shown in Fig 17. Through the analysis of the calculation results, we found that the unit impedance differences of most OD pairs are not significant, and only individual OD pairs have relatively high unit impedance. As can be seen from the figure, the impedance of the 18th, 19th, 25th, 36th, 37th, 40th, and 45th OD pairs is higher. Upon further in-depth analysis of these results, from the perspective of passengers’ departure preferences, for OD pairs with high impedance, it is likely that most of the passengers are commuters or on business trips, with relatively concentrated travel times, tending to depart during peak hours. This leads to relatively tight transportation resources during that period, thus increasing travel impedance. For example, during the morning rush hour on weekdays, a large number of commuters travel intensively, choosing limited train schedules within the same time period. This intensifies the competition faced by each passenger, increasing their travel time costs, congestion costs, etc., ultimately resulting in a higher travel impedance. Analyzing from the aspect of train service frequencies at different stations, the stations involved in high-impedance OD pairs may have relatively low train service frequencies. This means that passengers need to wait longer to board a train, and the increase in waiting time directly raises the time cost component in the generalized travel cost, thus increasing the overall travel impedance. For instance, at some remote area stations, due to relatively low passenger flow, the frequency of train stops is not high. When passengers travel between such stations, the proportion of waiting time in the total travel time is large, greatly increasing the travel impedance.

These analysis results are of great significance for railway operations. On the one hand, railway operation departments can optimize the lines and stations involved in high-impedance OD pairs based on these results. For example, increase the number of train trips during peak hours to alleviate the shortage of transportation resources; for stations with low service frequencies, reasonably adjust the train operation plan to increase the number of stops and reduce passengers’ waiting time, thereby reducing passengers’ travel impedance and enhancing the attractiveness of railway services. On the other hand, from a research perspective, follow-up studies can conduct more detailed investigations on these high-impedance OD pairs. Gather more detailed information about the passengers of these OD pairs, such as travel purposes, travel time distributions, and sensitivity to ticket prices, and establish more accurate models to simulate and predict the passenger flow changes of these OD pairs, so as to provide a more scientific basis for railway operation planning. Through in-depth exploration of these results, we can better understand the relationship between passengers’ travel behavior and railway transportation services, laying a more solid foundation for further research in this field.

## 6. Conclusion

Be directed against the different interest games between passengers and companies, a bi-level programming model of high-speed railway train timetable is established. The upper-level model is used to determine the departure, arrival, and stopping information of trains at each station and the unit fare of trains in each section and the number of EMUs with different seats in each train. The lower-level model is used to calculate the passenger flow distribution results. For the established bi-level programming model, a compound genetic algorithm with nested Frank-Wolfe method is designed. The effectiveness of the model and the algorithm is tested against the background of Lanzhou-Xi’an HSR. The computational results show that the method proposed in this paper can obtain a satisfactory high-speed train timetable with seating attributes within an acceptable solution time range.

In this paper, we mainly focus on the impact of fare and seat attributes on passenger travel, which in turn is used to optimize train timetables. Nevertheless, the train timetable problem is a very complex problem, and passengers’ preferences are also changing rapidly. Therefore, in future research, we should delve into the factors that affect passenger preferences and seek more robust timetables. Meanwhile, it is necessary to constantly search for more efficient models and algorithms, so as to realize not only highly fitting the timetable problem, but also solving it quickly, and conducting more refined research on the train timetable problem.

## Supporting information

S1 Table The daily average OD passenger flow from Lanzhou West to Xi’an North. By integrating the ticket selling information of relevant sections within a specific time period in the railway ticketing system, as well as the passenger flow data recorded by the passenger flow statistics equipment at the railway stations, such as (infrared sensing counters, video surveillance passenger flow analysis systems, etc.), and conducting sorting and averaging calculations, the data is obtained.
(DOCX)

S2 Table The values of distance (l_(i,j)) and running time (h_(i,j))of each section. This set of data may be sourced from the official railway line materials of the railway department to obtain the distances of each section. The running times might be collated from the actual operation records of trains or train timetables. They could also be accurately collected with the help of positioning and timing devices installed on the trains.
(DOCX)

S3 Table The values of other input parameters. Some parameters related to trains, vehicles and seats (such as the number of vehicles and seats, etc.) are derived from the basic operational data of railway departments; parameters involving costs (such as ticket prices and costs, etc.) are obtained by collecting actual operating ticket prices information and analyzing operational cost data; parameters related to time (such as intervals) are determined by referring to train operation scheduling plans and actual operation records; for other similar proportional parameters, they are set through statistical analysis of historical data of railway transportation systems or based on industry-wide common standards and empirical values.
(DOCX)

S4 Table Passengers’ preference Wa for departure time of OD pairs (1,j). Utilizing big data from railway ticketing systems, online travel platforms or other related travel data platforms. By analyzing the departure time distribution of passengers’ actual ticket purchases for different OD pairs, and calculating the proportion of ticket purchases in each time period, this can reflect passengers’ preferences for departure times and provide a basis for the table data.
(DOCX)
