# Supplementary material for: Optimizing the train timetable in a high-speed rail corridor: The implications on departure time, fare cost and seat preference of passengers
Source: PLoS One. 2025 Jun 18;20(6):e0326170. doi: 10.1371/journal.pone.0326170 (PMC12176190; doi:10.1371/journal.pone.0326170)
Supplement: S1 Table — By integrating the ticket selling information of relevant sections within a specific time period in the railway ticketing system, as well as the passenger flow data recorded by the passenger flow statistics equipment at the railway stations, such as (infrared sensing counters, video surveillance passenger flow analysis systems, etc.), and conducting sorting and averaging calculations, the data is obtained. (DOCX) [file pone.0326170.s001.docx]

**Table 7.** The daily average OD passenger flow from Lanzhou West to Xi’an North.

| Station $r$ | Station $s$ | | | | | | | | |
| --- | --- | --- | --- | --- | --- | --- | --- | --- | --- |
|  | 2 | 3 | 4 | 5 | 6 | 7 | 8 | 9 | 10 |
| 1 | 3371 | 1011 | 1686 | 5394 | 5057 | 1686 | 1349 | 1686 | 5730 |
| 2 | - | 450 | 393 | 674 | 450 | 168 | 168 | 225 | 843 |
| 3 | - | - | 113 | 168 | 113 | 56 | 56 | 113 | 225 |
| 4 | - | - | - | 225 | 113 | 56 | 56 | 168 | 225 |
| 5 | - | - | - | - | 1124 | 113 | 168 | 281 | 3933 |
| 6 | - | - | - | - | - | 563 | 843 | 843 | 2247 |
| 7 | - | - | - | - | - | - | 281 | 281 | 563 |
| 8 | - | - | - | - | - | - | - | 281 | 1124 |
| 9 | - | - | - | - | - | - | - | - | 281 |
| Total | 3371 | 1461 | 2192 | 6461 | 6855 | 2640 | 2919 | 3876 | 15170 |
