# Supplementary material for: Optimizing the train timetable in a high-speed rail corridor: The implications on departure time, fare cost and seat preference of passengers
Source: PLoS One. 2025 Jun 18;20(6):e0326170. doi: 10.1371/journal.pone.0326170 (PMC12176190; doi:10.1371/journal.pone.0326170)
Supplement: S2 Table — This set of data may be sourced from the official railway line materials of the railway department to obtain the distances of each section. The running times might be collated from the actual operation records of trains or train timetables. They could also be accurately collected with the help of positioning and timing devices installed on the trains. (DOCX) [file pone.0326170.s002.docx]

**Table 8.**The values of distance ($l_{i,j}$) and running time ($h_{i,j}$) of each section.

| Section ($i,j$) | 1-2 | 2-3 | 3-4 | 4-5 | 5-6 | 6-7 | 7-8 | 8-9 | 9-10 |
| --- | --- | --- | --- | --- | --- | --- | --- | --- | --- |
| Distance ($l_{i,j}$/km) | 103 | 78 | 55 | 41 | 141 | 37 | 41 | 60 | 35 |
| Running time ($h_{i,j}$/min) | 26.9 | 20.4 | 14.3 | 10.6 | 36.8 | 9.7 | 10.7 | 15.7 | 9 |
