# Supplementary material for: Optimizing the train timetable in a high-speed rail corridor: The implications on departure time, fare cost and seat preference of passengers
Source: PLoS One. 2025 Jun 18;20(6):e0326170. doi: 10.1371/journal.pone.0326170 (PMC12176190; doi:10.1371/journal.pone.0326170)
Supplement: S3 Table — Some parameters related to trains, vehicles and seats (such as the number of vehicles and seats, etc.) are derived from the basic operational data of railway departments; parameters involving costs (such as ticket prices and costs, etc.) are obtained by collecting actual operating ticket prices information and analyzing operational cost data; parameters related to time (such as intervals) are determined by referring to train operation scheduling plans and actual operation records; for other similar proportional parameters, they are set through statistical analysis of historical data of railway transportation systems or based on industry-wide common standards and empirical values. (DOCX) [file pone.0326170.s003.docx]

**Table 9.** The values of other input parameters.

| Parameter | Value | Parameter | Value | Parameter | Value |
| --- | --- | --- | --- | --- | --- |
| $n$ | 65/train | $\beta_{1}^{2}$ | 5 | $\varepsilon_{D}$ | 2/min |
| $n^{train}$ | 8/Vehicle | $\beta_{2}^{1}$ | 0.36 | $\varepsilon_{S}$ | 1/min |
| $\rho_{\min}^{section}$ | 3/min | $\beta_{2}^{2}$ | 0.4 | $E^{stop}$ | 400/CNY per min |
| $\rho_{\min}^{station}$ | 2/min | $\beta_{3}^{1}$ | 0.3 | $\theta_{\min}^{1}$ | 0.05 |
| $y_{\max}^{1}$ | 2/Vehicle | $\beta_{3}^{2}$ | 0.5 | $\theta_{\max}^{1}$ | 1 |
| $y_{\min}^{2}$ | 6/Vehicle | $\alpha$ | 2/CNY | $\theta_{\min}^{2}$ | 0.4 |
| $y_{\min}^{1}$ | 1/Vehicle | $\gamma$ | 2/CNY per min | $\theta_{\max}^{2}$ | 1 |
| $y_{\max}^{2}$ | 7/Vehicle | $E_{1}^{car}$ | 4500/CNY | $g_{\min}^{1}$ | 0.35/CNY per person-kilometer |
| $D_{1}$ | 60/seat | $E_{2}^{car}$ | 3700/CNY | $g_{\max}^{1}$ | 0.5/CNY per person-kilometer |
| $D_{2}$ | 80/seat | $\mu_{\max}$ | 6/min | $g_{\min}^{2}$ | 0.2/CNY per person-kilometer |
| $\beta_{1}^{1}$ | 3 | $\mu_{\min}$ | 3/min | $g_{\max}^{2}$ | 0.4/CNY per person-kilometer |
