# Supplementary material for: Optimizing the train timetable in a high-speed rail corridor: The implications on departure time, fare cost and seat preference of passengers
Source: PLoS One. 2025 Jun 18;20(6):e0326170. doi: 10.1371/journal.pone.0326170 (PMC12176190; doi:10.1371/journal.pone.0326170)
Supplement: S4 Table — Utilizing big data from railway ticketing systems, online travel platforms or other related travel data platforms. By analyzing the departure time distribution of passengers’ actual ticket purchases for different OD pairs, and calculating the proportion of ticket purchases in each time period, this can reflect passengers’ preferences for departure times and provide a basis for the table data. (DOCX) [file pone.0326170.s004.docx]

**Table 10.** Passengers’ preference $w_{a}$ for departure time of OD pairs $(1,j)$.

| Time-period (by hours) | OD pairs | | | | | | | | |
| --- | --- | --- | --- | --- | --- | --- | --- | --- | --- |
|  | 1-2 | 1-3 | 1-4 | 1-5 | 1-6 | 1-7 | 1-8 | 1-9 | 1-10 |
| 1 | 0.29 | 0.40 | 0.42 | 0.26 | 0.25 | 0.40 | 0.25 | 0.41 | 0.50 |
| 2 | 0.31 | 0.32 | 0.47 | 0.42 | 0.45 | 0.35 | 0.51 | 0.50 | 0.57 |
| 3 | 0.66 | 0.73 | 0.46 | 0.62 | 0.51 | 0.72 | 0.65 | 0.56 | 0.71 |
| 4 | 0.64 | 0.65 | 0.49 | 0.75 | 0.61 | 0.61 | 0.61 | 0.55 | 0.72 |
| 5 | 0.78 | 0.70 | 0.82 | 0.74 | 0.60 | 0.66 | 0.72 | 0.63 | 0.62 |
| 6 | 0.60 | 0.61 | 0.66 | 0.63 | 0.73 | 0.65 | 0.64 | 0.71 | 0.45 |
| 7 | 0.64 | 0.53 | 0.62 | 0.43 | 0.42 | 0.54 | 0.41 | 0.62 | 0.56 |
| 8 | 0.56 | 0.38 | 0.62 | 0.47 | 0.52 | 0.55 | 0.38 | 0.66 | 0.54 |
| 9 | 0.59 | 0.31 | 0.55 | 0.57 | 0.36 | 0.36 | 0.44 | 0.42 | 0.43 |
| 10 | 0.54 | 0.45 | 0.49 | 0.64 | 0.48 | 0.43 | 0.62 | 0.36 | 0.36 |
| 11 | 0.42 | 0.43 | 0.45 | 0.68 | 0.62 | 0.44 | 0.61 | 0.57 | 0.53 |
| 12 | 0.39 | 0.58 | 0.46 | 0.73 | 0.53 | 0.61 | 0.37 | 0.47 | 0.56 |
| 13 | 0.59 | 0.69 | 0.54 | 0.58 | 0.68 | 0.67 | 0.67 | 0.75 | 0.53 |
| 14 | 0.67 | 0.50 | 0.43 | 0.38 | 0.65 | 0.50 | 0.69 | 0.56 | 0.64 |
| 15 | 0.40 | 0.59 | 0.49 | 0.32 | 0.54 | 0.64 | 0.42 | 0.47 | 0.55 |
| 16 | 0.35 | 0.46 | 0.48 | 0.29 | 0.43 | 0.43 | 0.55 | 0.46 | 0.28 |
| 17 | 0.36 | 0.38 | 0.34 | 0.27 | 0.35 | 0.27 | 0.29 | 0.31 | 0.51 |
| 18 | 0.11 | 0.17 | 0.18 | 0.21 | 0.16 | 0.13 | 0.18 | 0.21 | 0.15 |
